# Solid State Room Temperature Dual Phosphorescence from 3-(2-Fluoropyridin-4-yl)triimidazo[1,2-*a*:1′,2′-*c*:1″,2″-*e*][1,3,5]triazine

**DOI:** 10.3390/molecules24142552

**Published:** 2019-07-13

**Authors:** Andrea Previtali, Elena Lucenti, Alessandra Forni, Luca Mauri, Chiara Botta, Clelia Giannini, Daniele Malpicci, Daniele Marinotto, Stefania Righetto, Elena Cariati

**Affiliations:** 1 Department of Chemistry, Università degli Studi di Milano and INSTM RU, via Golgi 19, 20133 Milano, Italy; 2 Institute of Molecular Science and Technologies (ISTM) of CNR and INSTM RU, via Golgi 19, 20133 Milano, Italy; 3 Institute for Macromolecular Studies (ISMAC) of CNR, via Corti 12, 20133 Milano, Italy

**Keywords:** room temperature ultralong phosphorescence, organic phosphorescence, time resolved spectroscopy, H aggregation

## Abstract

Organic room temperature persistent luminescence is a fascinating but still largely unexplored phenomenon. Cyclic-triimidazole and its halogenated (Br, I) derivatives have recently revealed as intriguing phosphors characterized by multifaceted emissive behavior including room temperature ultralong phosphorescence (RTUP) associated with the presence of H-aggregates in their crystal structure. Here, we move towards a multicomponent system by incorporating a fluoropyridinic fragment on the cyclic-triimidazole scaffold. Such chromophore enhances the molecular properties resulting in a high photoluminescence quantum yield (PL QY) in solution but preserves the solid-state RTUP. By means of X-ray diffraction (XRD) analysis, theoretical calculations, steady-state and time-resolved spectroscopy on solutions, polymethylmethacrylate (PMMA) blends and crystals, the nature of the different radiative deactivation channels of the compound has been disclosed. In particular, the molecular fluorescence and phosphorescence, this latter observed in frozen solution and in PMMA blends, are associated to deactivation from S_1_ and T_1_ respectively, while the low energy RTUP, observed only for crystals, is interpreted as originated from H aggregates.

## 1. Introduction

Luminophores, characterized in the solid-state by prolonged emission after excitation removal with lifetime longer than 0.1 s, are receiving great attention in the recent years [1,2], due to their applications in emerging technologies spanning from anti-counterfeiting [3,4], bio-imaging [5,6], lighting and display [7]. 

Compared to well-developed inorganic counterparts, organic materials showing room temperature ultralong phosphorescence (RTUP) offer many advantages such as lower toxicity and higher structural versatility. 

Enhancing the efficiency of RTUP while maintaining long lifetimes is a great challenge, due to the presence of multiple competitive decay channels. In this regard, the most evident role of crystal packing is that of suppressing molecular motions and protecting from oxygen quenching. However, more specific intermolecular interactions (H-aggregation, halogen and hydrogen bonding) can be decisive in activating RTUP [8,9,10,11,12,13,14,15]. In particular, the mechanism of RTUP in H-aggregated compounds relies on the population of triplet excitons via intersystem crossing (ISC) from photoexcited singlet excitons (usually S_1_), followed by H-aggregates exciton trapping and radiative relaxation of the trapped triplet excitons to the ground state (S_0_). Fast singlet-triplet ISC occurs when both the energy gap law and El-Sayed rule are satisfied.

We have recently reported on the intriguing photophysical behavior of triimidazo [1,2-*a*:1′,2′-*c*:1″,2″-*e*][1,3,5]triazine (TT) [9], and its Br- and I-derivatives [16,17,18], where exciton trapping by H aggregates provides an effective means of stabilizing and protecting the triplet excitons formed through intersystem crossing [9]. In particular, TT is characterized by crystallization-induced and mechanochromic emissive behavior, as well as RTUP (1 s) at ambient conditions. The presence of one or multiple heavy (Br and I) atoms on the TT scaffold greatly modifies both its molecular and solid-state photophysical behavior resulting in a complex photoluminescence (PL) with emissions going from dual fluorescence (2F), molecular phosphorescence (MP) to supramolecular room temperature phosphorescence (RTP) and RTUP [16,17,18].

Here, a step forward in the chemistry and photophysics of TT-derivatives is accomplished by insertion of a chromophoric fragment (2-fluoropyridine) on the trimidazolic scaffold. Organic substituents are expected to modify the emissive properties at both molecular and, through different packing arrangement, solid-state levels. Unfortunately, these effects are not predictable at this stage, so that any new member of the TT-family represents a building block worth studying to get information on this new and very intriguing class of emitters.

## 2. Results

**Scheme 1:**
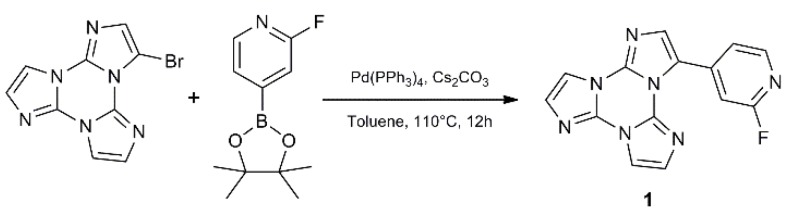
Synthesis of **1**.

3-(2-fluoropyridin-4-yl)triimidazo[1,2-*a*:1′,2′-*c*:1″,2″-*e*][1,3,5]triazine, **1**, containing the 2-fluoropyridine moiety, has been synthetized by Suzuki-Miyaura coupling between 3-bromo-triimidazo[1,2-*a*:1′,2′-*c*:1″,2″-*e*][1,3,5]triazine and 2-fluoropyridine-4-boronic acid pinacol ester (Scheme 1).

**Figure 1:**
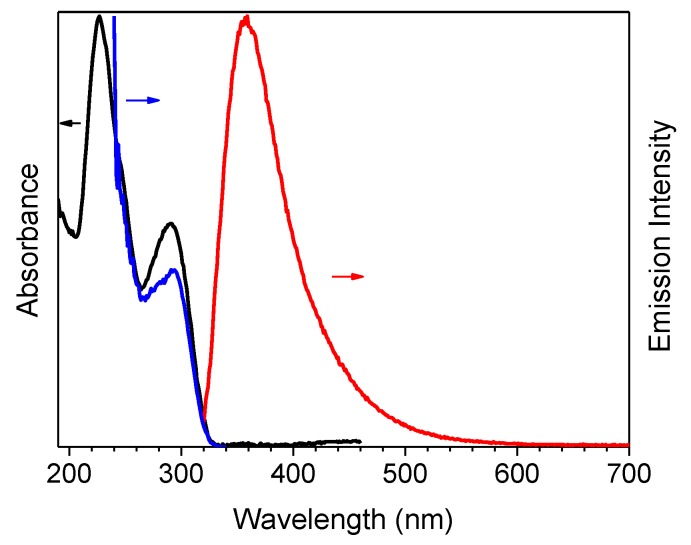
**1** in CH_3_CN (10^−5^ M) at 298 K: normalized absorption (black line), emission (λ_exc_ = 300 nm, red line) and excitation spectra (λ_em_ = 358 nm, blue line).

The compound’s photophysical behavior is markedly different from that of parent TT being, already as a molecule, quite fluorescent at room temperature and showing additional molecular phosphorescence at 77 K. In fact, solutions of **1** in CH_3_CN (10^−5^ M) display at 298 K absorption maxima at 227 and 291 nm, and an emission band at 358 nm (Φ = 50 %), corresponding to radiative S_1_-S_0_ deactivation (τ = 4.26 ns, see Table 1 and Figure 1). Absorption and emission spectra in solvents of different polarity (see Figure S7) reveal a weak positive solvatochromism for the low energy band and a negative one for the high energy band (Δλ = 3 and 17 nm, respectively, from CHCl_3_ to CH_3_CN).

**Figure 2:**
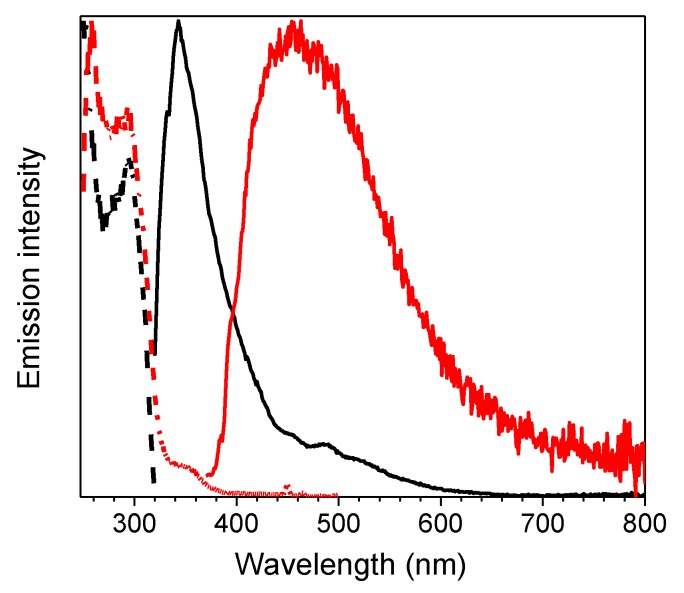
**1** in CH_3_CN (10^−5^ M) at 77 K: normalized emission (λ_exc_ = 300 nm, black line; λ_exc_ = 350 nm, red line) and excitation (λ_em_ = 343 nm, black dashed line; λ_em_ = 450 nm, red dashed line) spectra.

At 77 K, a slight blue shift (344 nm, τ_av_ = 4.05 ns) of the fluorescent emission was observed by exciting at 300 nm. Interestingly, at this low temperature, a weak phosphorescent low energy tail appeared in the PL spectrum and could be isolated (454 nm, τ_av_ = 1.63 s) from the fluorescent component by selectively populating the T_1_ level (λ_exc_ = 350 nm) (see Table 1 and Figure 2). On this regard, the importance of direct S_0_-T_1_ excitation of organic phosphorescent compounds has been highlighted by Huang and coworkers [19].

**Figure 3:**
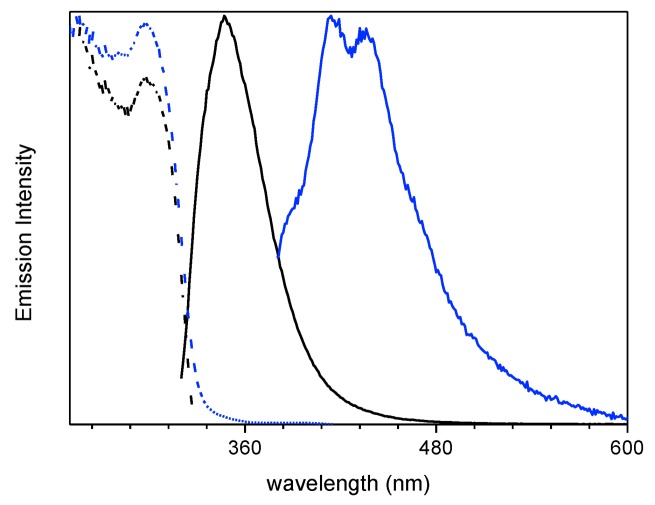
6 wt.% loading **1**:PMMA film at 298 K: normalized emission (λ_exc_ = 300 nm, black line; λ_exc_ = 360 nm, blue line) and excitation (λ_em_ = 348nm, black dashed line; λ_em_ = 436 nm, blue dashed line) spectra.

Spin coated thin film of **1** dispersed in polymethylmethacrylate (PMMA) (6 wt.%) displayed at 298 K an intense fluorescent emission at 348 nm (τ_av_ = 1.89 ns) together with a weak phosphorescent component which can be isolated (415, 436 nm, τ_av_ = 3.09 ms) from the fast emission by exciting at low energy (λ_exc_ = 360 nm) (see Table 1 and Figure 3). 

**Figure 4:**
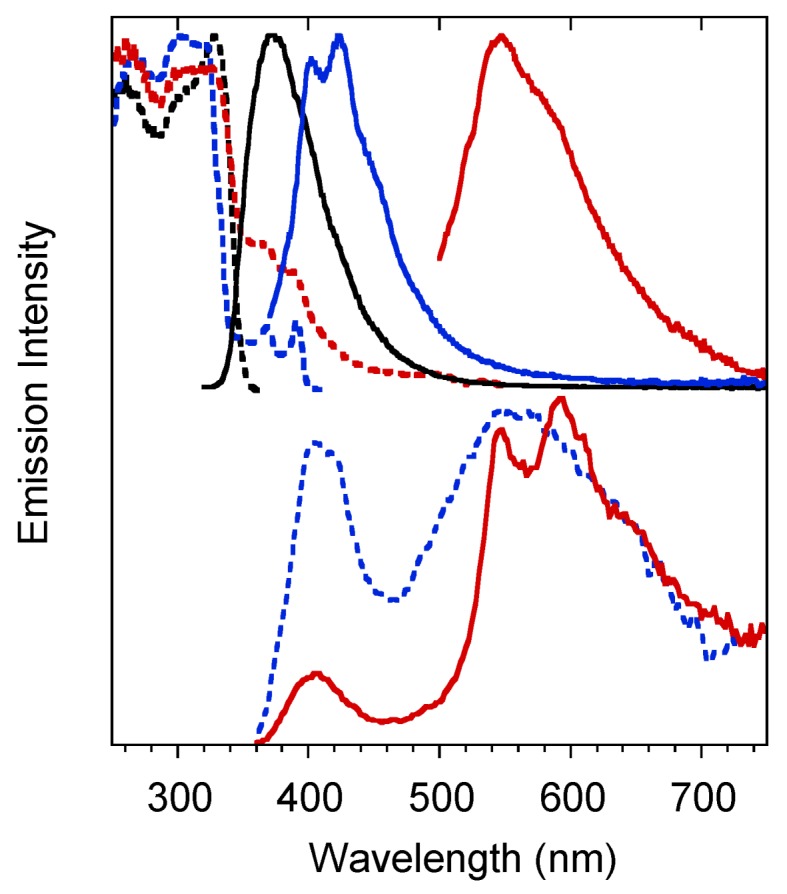
Crystals of **1** at 298 K. Upper panel: normalized emission (λ_exc_ = 300 nm, black solid line; λ_exc_ = 360 nm, blue solid line; λ_exc_ = 480 nm, red solid line) and excitation (λ_em_ = 373 nm, black dashed line; λ_em_ = 425 nm, blue dashed line; λ_em_ = 570 nm, red dashed line) spectra. Bottom panel: normalized phosphorescence spectra (λ_exc_ = 300 nm; delay 200 μs, window 1 ms, blue dashed line; delay 5 ms, window 20 ms, red solid line).

Crystals of **1** are characterized by multiple emissions. In particular, at 298 K, a fluorescent (373 nm, τ_av_ = 4.77 ns) and two phosphorescent (403, 424 and 446 nm, τ_av_ = 11.64 ms; 547 nm, τ_av_ = 417.78 ms) bands, with overall quantum efficiency equal to 25%, were observed in the PL spectrum by exciting at 300, 360 and 480 nm respectively (see Figure 4 and Table 1). The two phosphorescences were separated from the prompt emission in the delayed spectra (see Figure 4 bottom) where the longest-lived component displays peaks at 546 and 592nm.

**Figure 5:**
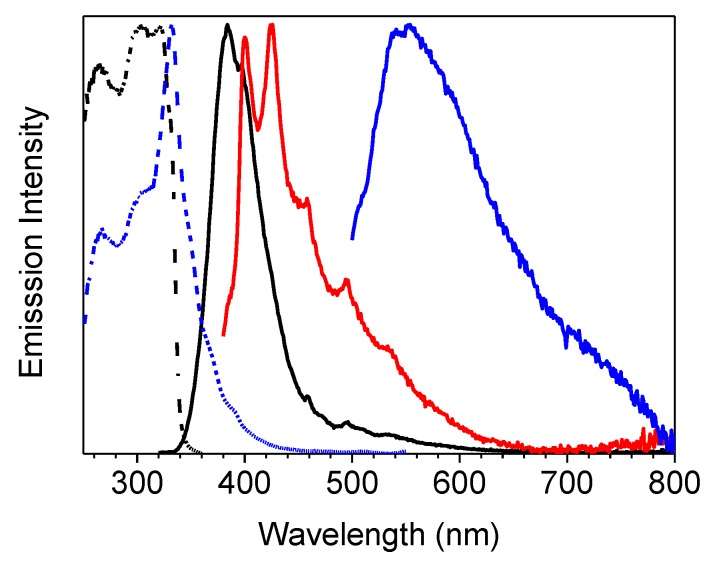
Crystals of **1** at 77 K: normalized emission (λ_exc_ = 300 nm, black line; λ_exc_ = 360 nm, red line; λ_exc_ = 480 nm, blue line) and excitation (λ_em_ = 375 nm, black dashed line; λ_em_ = 570 nm, blue dashed line line) spectra.

At 77 K the three emissions are still visible in almost the same position (see Figure 5 and Table 1), but, for the lower energy phosphorescent band, much longer lifetimes are measured (up to 2s).

Both phosphorescent emissions display vibronic replicas with energy separation (about 180 meV) that can be associated to a vibronic progression involving the imidazole ring modes [20].

## 3. Discussion

**Figure 6:**
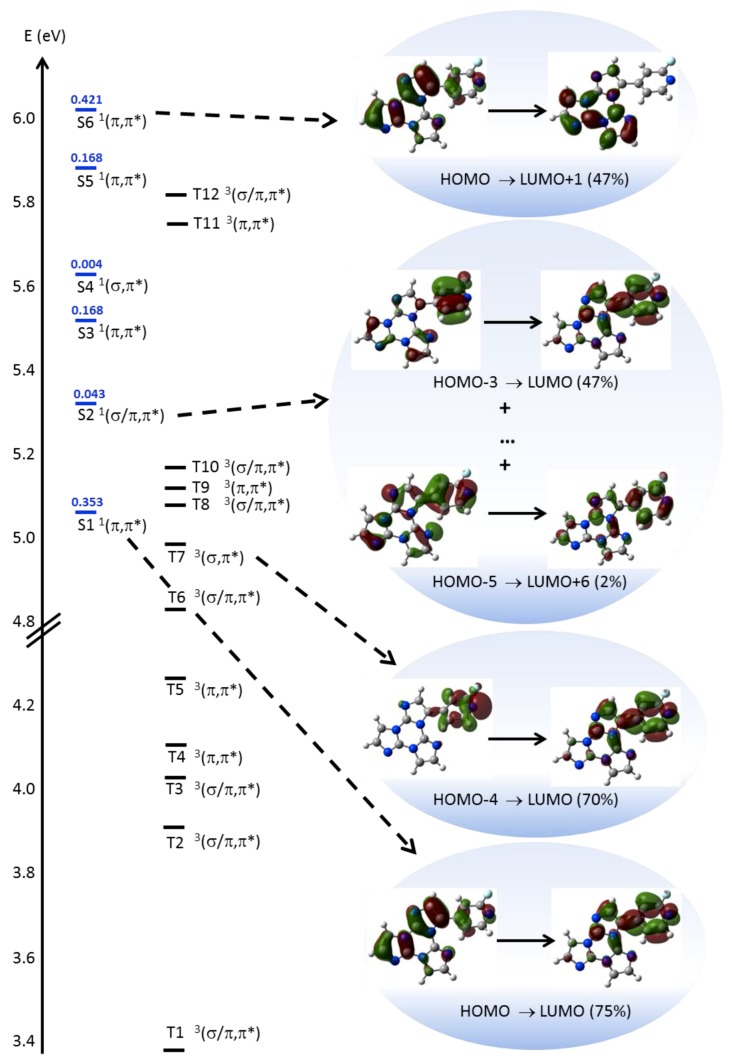
Electronic levels computed for **1** (**left**) and selected MO involved in the transitions (**right**) at molecular level. In blue are reported the singlet levels with the corresponding oscillator strengths *f.*

In order to interpret the photophysical behavior of **1**, theoretical calculations and single-crystal X-ray diffraction analysis have been performed. DFT geometry optimization of **1**, starting from its X-ray molecular structure (see below), was first performed to investigate the behavior of the luminophor in solution. Owing to the possible rotation around the single bond connecting the two chromophores, the occurrence of other local minima, which can be present in solution besides that observed in the crystal, has been then tested (see Figure S22). Due to the asymmetry generated by the fluorine substitution, two independent and almost isoenergetic minima are present in the potential energy surface, besides the symmetric ones with respect to the TT least squares (l.s.) plane. Except for the different positions of the fluorine atom, the two conformations are quite similar, as denoted by the dihedral angle between the l.s. planes through TT and 2-fluoropyridine, measuring 47° in the X-ray derived geometry and 43° in the additional minimum. The rather twisted conformations suggest a reduced conjugation between the aromatic moieties within the molecule. Moreover, the presence of the fluorine atom grants some degree of polarity to the molecule (μ_g_ = 4.03 and 3.75 D in the two conformations, respectively). TDDFT calculations on **1** in the two optimized conformations provide very similar results, i.e., for the more stable one (see SI for the second minimum), a strong π→π* transition (S_0_→S_1_ at 245 nm, f = 0.353, see Figure 6), dominated by the HOMO→LUMO contribution with charge transfer (CT) character (from TT to 2-fluoropyridine), in the same direction as the ground state dipole moment), followed by a weak one (S_0_→S_2_ at 234 nm, f=0.043) of mixed σ/π→π* character. One of the occupied MOs involved in the latter (HOMO-5, see Figure 6 and Figure S23) is, in fact, a π orbital except for a σ contribution localized on a TT nitrogen atom. 

Then a series of π→π* transitions are computed, whose envelope well reproduces the absorption band observed at high energy. Among these transitions, the stronger one (S_0_→S_6_ at 205 nm, f = 0.421) is dominated by the HOMO→LUMO+1 contribution having CT character from 2-fluoropyridine to TT, i.e. in the opposite direction with respect to S_0_→S_1_. Accordingly, the computed Δμ_eg_ = μ_e_ − μ_g_ (being μ_e_ the excited state dipole moment) is almost vanishing (0.24 D) for S_0_→S_6_, unlike that of S_0_→S_1_ (2.88 D). Accordingly, additional IEFPCM (TD)DFT calculations on **1** in chloroform and acetonitrile, providing dipole moments equal to 4.75 and 5.03 D, respectively, result into even more evident discrepancies between the values of Δμ_eg_ for the two transitions (4.68 and 5.47 D for S_0_→S_1_; −1.74 and −2.32 D for S_0_→S_6_, in chloroform and acetonitrile respectively). Such results support the positive and the negative solvatochromisms as observed for the low and high energy absorption bands, respectively, in different solvents. It should be mentioned, however, that IEFPCM calculations are able to reproduce only the positive solvatochromism (by about 3 nm) of the low energy band. The high energy band, resulting from the convolution of several transitions besides S_0_→S_6_, does not undergo essentially any shift by changing the solvent, probably owing to a too low weight of S_0_→S_6_, with respect to the other high energy excitations. Such a result is a consequence of the well-known difficulty of TDDFT to correctly describe CT excited states.

As far as the molecular phosphorescent emission observed at ca. 450 nm in frozen solution, TDDFT calculations suggest that it can be associated with the presence of a T_n_ level (at 250 nm, T_7_ in Figure 6) with (σ,π*) symmetry (where the occupied orbital, HOMO-4, is localized on pyridine) that allows fast ISC from the close S_1_ (π,π*) level (computed at 245 nm) according to El-Sayed rule. Phosphorescence from T_1_ then occurs after internal conversion (IC) from T_n_. Its molecular origin is confirmed by the presence of a long-lasting component at similar energy (ca. 430 nm) in spin-coated PMMA blend at room temperature. 

In agreement to what observed in solution and PMMA blends, the fluorescent and the high energy phosphorescent bands of the crystals can be assigned to radiative deactivation from S_1_ and T_1_ molecular levels, respectively. The additional phosphorescent emission is not recognizable as a molecular contribution and to disclose its origin, we have investigated the single-crystal structure of **1**.

**Figure 7:**
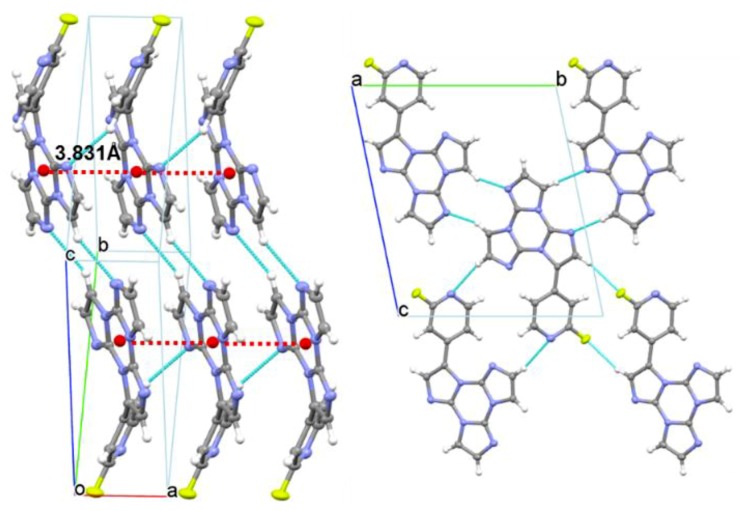
Partial views along bc (**left**) and a directions (**right**) of **1** crystal structure showing columnar H-aggregates (centroids of the triazinic rings shown as red circles) and hydrogen bonds (light blue dashed lines).

Compound **1** crystallizes in the P-1 space group, forming infinite columns of equidistant face-to-face stacked molecules along the crystallographic axis (see Figure 7). Along the column axis, molecules are iso-oriented and placed at interplanar distances equal to 3.366 Å with a rather small slippage (1.9 Å), short distances between centroids of triazinic rings (3.831 Å) and high angle θ (61°) between the centroid-centroid vector and the projection of this vector on the molecular plane. Such stacking features are indicative of H-aggregation in the structure of **1**, though some differences with respect to the stacking pattern of the parent TT structure [21] should be evidenced. The latter, in fact, shows infinite ABAB… alternating stacks where each molecule is rotated by 180°, with respect to the adjacent ones, about an axis normal to the molecular l.s. planes, with alternating distances between average molecular planes of 3.204 and 3.290 Å and centroid-centroid distances of 3.733 and 3.949 Å. The π-π stacking mode of **1** is instead quite similar to that of the monoiodo-triimidazole derivative [18], forming infinite columns of iso-oriented and equidistant face-to-face stacked molecules, though with larger slippage (2.3 Å, θ = 55°) and longer distance between centroids of triazinic rings (4.097 Å). Molecules of **1** are laterally connected through several C-H···N and C-H···F close contacts (the shorter one being C8-H8···N1, H···N = 2.52 Å, C-H···N = 170.5°) forming slightly corrugated planes. The dihedral angle between l.s. planes through TT and pyridine moieties measures 49.80°. This value, close to that computed for the isolated molecule (47.00°), suggests minor conformational rearrangement from solution to the solid-state. 

**Figure 8:**
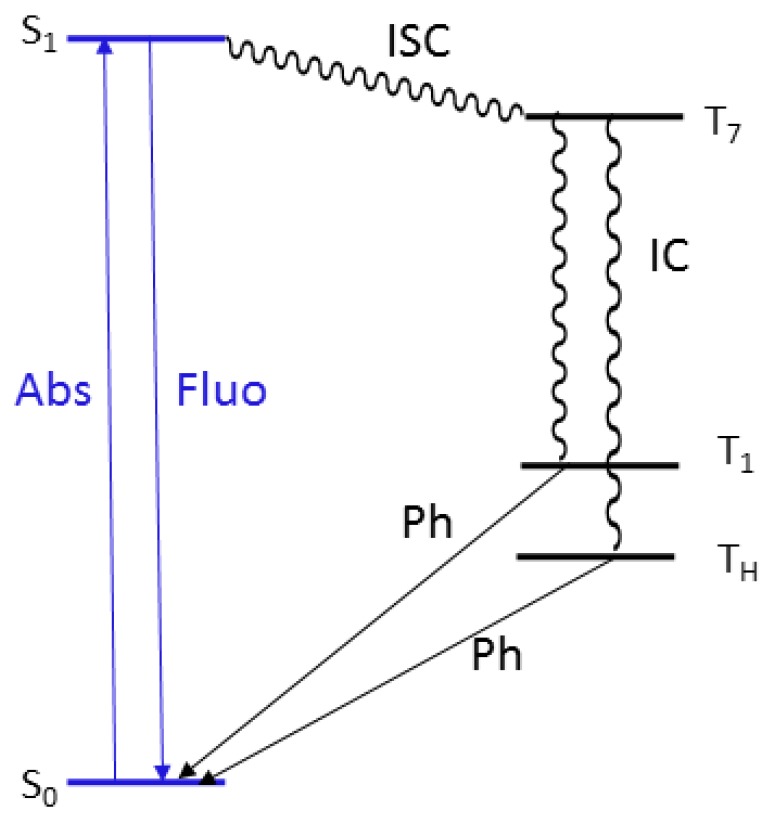
Energy level diagrams showing transitions associated with fluorescence (blue) and phosphorescence (black).

This crystal structure justifies both phosphorescent emissions (see Jablonski diagram reported in Figure 8). The molecular (high energy) component, visible in solution only at 77 K, appears at 298 K in the solid due to the rigidifying effect and protection from oxygen quenching ascribable to intermolecular interactions, and, on the other hand, the low energy phosphorescence can be associated to the presence of H aggregates in the crystal structure, in agreement with previous findings on compounds with the same triazinic scaffold [9,16,17,18]. This interpretation is in line to the results obtained from the PMMA blend in which the molecular phosphorescence is visible at 298 K thanks to the rigidifying effect of the PMMA matrix, while the low energy component is lacking due to the absence of supramolecular organization of the phosphor into H aggregates. 

In summary, we have isolated and characterized a new member of the photophysically intriguing TT family where the presence of a fluoropyridine fragment provides increased molecular performances, comprising fluorescence and heavy atom free phosphorescence, still preserving solid-state RTUP. This work adds a new building block to the knowledge of this family and to RTP organic phenomena in general. From these results, the preparation of new multicomponent emitters can be envisaged. 

## 4. Materials and Methods 

### 4.1. General Information

All reagents and solvents were purchased from Merck (Darmstadt, Germany) and used without further purification unless otherwise stated. 3-bromo-triimidazo[1,2-*a*:1′,2′-*c*:1″,2″-*e*][1,3,5]triazine was prepared according to literature procedures [17]. ^1^H and ^13^C-NMR spectra were recorded on a Bruker (Bruker Italia S.r.l. Milano, Italy) AVANCE-400 instrument (400 MHz), ^19^F-NMR spectra were recorded on Bruker AVANCE DRX-300 instrument (300 MHz). Chemical shifts are reported in parts per million (ppm) and are referenced to the residual solvent peak (DMSO, ^1^H 2.5 ppm, ^13^C 39.5 ppm) and to CFCl_3_ for ^19^F resonances. Coupling constants (*J*) are given in hertz (Hz) and are quoted to the nearest 0.5 Hz. Peak multiplicities are described in the following way: s, singlet; d, doublet, m, multiplet. Mass spectra were recorded on a Thermo Fisher (Thermo Fisher Scientific, Waltham, MA USA) LCQ Fleet Ion Trap Mass Spectrometer equipped with UltiMate™ 3000 HPLC system. UV-Visible spectra were collected by a Shimadzu (Shimadzu Italia S.r.l., Milano, Italy) UV3600 spectrophotometer. Photoluminescence quantum yields were measured using a C11347 Quantaurus–Absolute Photoluminescence Quantum Yield Spectrometer (Hamamatsu Photonics K.K, Arese (MI), Italy), equipped with a 150 W Xenon lamp, an integrating sphere and a multichannel detector. Steady-state emission and excitation spectra and photoluminescence lifetimes were obtained using an FLS 980 (Edinburg Instrument Ltd, Livingston, UK) and a Nanolog (Horiba Scientific, Piscataway, NJ, USA) spectrofluorimeter. The steady-state measurements were recorded by a 450 W Xenon arc lamp. Photoluminescence lifetime measurements were performed using: Edinburgh Picosecond Pulsed Diode Laser EPL-375, EPLED-300, (Edinburg Instrument Ltd, Livingston, UK) and microsecond flash Xe-lamp (60 W, 0.1 ÷ 100 Hz) with data acquisition devices time-correlated single-photon counting (TCSPC) and multi-channel scaling (MCS) methods, respectively. Average lifetimes are obtained as τ_av_ = $\frac{\sum A_{i}\tau_{i}^{2}}{\sum A_{i}\tau_{i}}$ from bi-exponential or three-exponential fits. Low temperature measurements are performed by immersion of the sample in an LN_2_ quartz dewar or with a variable temperature liquid nitrogen cryostat Oxford DN1704.

Compound **1** was obtained by Suzuki-Miyaura coupling between 3-bromo-triimidazo[1,2-*a*:1′,2′-*c*:1″,2″-*e*][1,3,5]triazine and 2-fluoropyridine-4-boronic acid pinacol ester. The reaction was performed under nitrogen in a Schlenk flask. 3-bromo-triimidazo[1,2-*a*:1′,2′-*c*:1″,2″-*e*][1,3,5]triazine (300 mg, 1.08 mmol), 2-fluoropyridine-4-boronic acid pinacol ester (340 mg, 1.52 mmol), cesium carbonate (1.76 g, 5.40 mmol), tetrakis(triphenylphosphine)palladium(0) Pd(PPh_3_)_4_ (120 mg, 0.10 mmol) and anhydrous toluene (10 mL) were transferred inside the Schlenk flask. The mixture was heated at 110 °C under static nitrogen for 12 h. The reaction was then cooled to room temperature, diluted with CH_2_Cl_2_ (80 mL), filtered on buchner and evaporated to dryness. The crude product was washed with hexane to remove Ph_3_PO (identified by ^1^H, ^31^P-NMR and mass spectroscopy) and then purified by column chromatography with AcOEt on silica gel (R_f_ = 0.23) to give pure **1** (190 mg, yield 60%). Crystals suitable for X-ray diffraction studies were obtained by layering a CH_2_Cl_2_ solution of **1** with hexane. Anal. Found. (calcd.): C, 57.18 (57.34); H, 2.61 (2.75); N, 33.53 (33.43).

Films of **1** dispersed in polymethylmethacrylate (PMMA) were prepared by spin coating (2000 rpm, 60 s) a dichloromethane solution (**1**/PMMA = 6 wt%; PMMA = 10 wt% with respect to the solvent) on a glass substrate.

### 4.2. Single Crystal X-Ray Studies

X-ray data of **1** were collected on a Bruker Apex II diffractometer (Bruker AXS Inc., Madison, WI, USA) using MoKα radiation [22]. The structure was solved using direct methods and refined with SHELXL-14 [23] using a full-matrix least squares procedure based on F^2^ using all data. Hydrogen atoms were placed at geometrically estimated positions. Details relating to the crystal and the structural refinement are presented in Table S1. Rather thin colorless tablets of **1** were grown at room temperature by adding hexane to a CH_2_Cl_2_ solution of the compound. Full details of crystal data and structure refinement, in CIF format, are available as Supplementary Information. CCDC reference number: 1913040.

### 4.3. Computational Details

DFT and TDDFT calculations on monomeric **1** were performed with Gaussian 16 program (Revision A.03) [24] using the 6-311++G(d,p) basis set. Its geometry has been optimized starting from the experimental molecular structure as derived from X-ray studies, both in vacuo and in solvent (chloroform and acetonitrile) through the IEFPCM approach [25]. Scan calculations have been then performed by varying a torsion angle connecting fluoropyridine with the TT moiety. For comparison purposes, we have adopted the same functional ωB97X [26] as used for calculations on the previously reported parent cyclic triimidazole and its halogenated derivatives.

## Figures and Tables

**Table 1:** Photoluminescence data of **1** at 298 and 77 K.

Sample	298 K					77 K			
	Φ (%)	λ_abs_^1^ (nm)	λ_em_ (nm)	τ (λ_exc_ 300nm)	Origin	λ_abs_^1^ (nm)	λ_em_ (nm)	τ (λ_exc_ 300nm)	Origin
**1** (CH_3_CN)	50	290	358	4.26ns ^2^	S_1_-S_0_	295	344	0.44 ns (0.52), 1.74 ns (0.40) 7.9 ns (0.08) ^3^	S_1_-S_0_
						350	454	127 ms (0.07), 1640 ms (0.93) ^4^	T_1_-S_0_
**1** (crystals)	25	301	373	1.78 ns (0.19), 5.02 ns (0.81) ^5^	S_1_-S_0_	306	385	7.56 ns (0.10), 16.87 ns (0.90) ^5^	S_1_-S_0_
		368, 392	403, 424, 446	1.26 ms (0.40), 12.35 ms (0.60) ^6^	T_1_-S_0_	369, 390	401, 425, 457	1.52 ms (0.45), 13.97 ms (0.55) ^6^	T_1_-S_0_
		500, 533	546 592	42.27 ms (0.38), 441.21 ms (0.62) ^7^	$T_{1}^{H}$-S_0_	513	549	175.95 ms (0.03), 1190.09 ms (0.49)2320.45 ms (0.48)^7^	$T_{1}^{H}$-S_0_
**1**(film)		298	348	0.84 ns (0.28), 2.06 ns (0.72) ^8^	S_1_-S_0_				
			415, 436	0.57 ms (0.50), 3.50 ms (0.50) ^9^	T_1_-S_0_				

^1^ taken from excitation spectra; ^2^ λ_em_ = 358 nm; ^3^ λ_em_ = 343 nm; ^4^ λ_em_ = 450 nm; ^5^ λ_em_ = 373 nm; ^6^ λ_em_ = 423 nm; ^7^ λ_em_ = 547 nm; ^8^ λ_em_ = 347 nm; ^9^ λ_em_ = 453 nm.

## Supplementary Materials

The following are available online, Figures S1–S5: ^1^H, ^19^F and ^13^C-NMR Spectra; Figure S6: HPLC-MS profile; Figure S7: Absorption Spectra; Figures S8–S18: Emission decay plots; Figure S19: Ortep view with labelling scheme. Ellipsoids at 50% level of probability; Figure S20: ωB97X/6-311++G(d,p) computed absorption spectrum of optimized **1** resulting from convolution of the excitation energies (blue sticks) with 0.25 eV of half-bandwidth; Figure S21: Electronic levels computed for TT (left) and **1** (right) at molecular level. In blue are reported the singlet levels with oscillator strength *f* ≥ 0.001 and the corresponding values of *f*; Figure S22: Scan of total energy as a function of the N2C2C10C14 torsion angle; Figure S23: Isodensity surface plot of the frontier orbitals of **1** mainly involved in the computed transitions (isosurface values: 0.03, energies in a.u); Figure S24: Cyclic voltammetry patterns for **1**; Table S1: Crystallographic data and structure refinement details; Table S2: First TD-ωB97X/6-311++G(d,p) S_0_→S_n_ and T_0_→T_n_ transitions computed for **1**; Table S3: Selected cyclic voltammetry data for **1** on GC electrode.
