# Supplementary material for: Solid State Room Temperature Dual Phosphorescence from 3-(2-Fluoropyridin-4-yl)triimidazo[1,2-a:1′,2′-c:1″,2″-e][1,3,5]triazine
Source: Molecules. 2019 Jul 13;24(14):2552. doi: 10.3390/molecules24142552 (PMC6680853; doi:10.3390/molecules24142552)

Solid State Room Temperature Dual Phosphorescence from 3-(2-fluoropyridin-4-yl)triimidazo[1,2-*a*:1',2'-*c*:1'',2''-*e*][1,3,5]triazine

Andrea Previtali,[a,b] Elena Lucenti,[b] Alessandra Forni,*[b] Luca Mauri,[a] Chiara Botta,[c] Clelia Giannini,[a] Daniele Malpicci,[a] Daniele Marinotto,[b] Stefania Righetto,[a,b] and Elena Cariati*[a,b]

NMR data of 1 (9.4 T, DMSO-d$_6$, 298 K, δ, ppm)

^{1}H NMR 8.32 (1H, d, *J* = 5.3 Hz), 8.02 (2H, m), 7.82 (1H, m), 7.75 (1H, s), 7.71 (1H, m), 7.33 (1H, d, *J* = 1.7 Hz), 7.29 (1H, d, *J* = 1.6 Hz);

^{13}C NMR 163.0 CF, (1C, d, *J*= 233.3 Hz), 147.1 CH (1C, d, *J* = 14.7 Hz), 141.2 C (1C, d, *J* = 8.8 Hz), 138.1 C (1C, s), 135.9 C (1C, s), 135.6 C (1C, s), 130.8 CH, (1C, s), 128.8 CH (1C, s), 127.9 CH (1C, s), 124.6 C (1C, d, *J* = 4.4 Hz), 121.4 CH (1C, d, *J* = 2.9 Hz), 111.9 CH (1C, s), 111.5 CH (1C, s), 108.5 CH (1C, d, *J* = 39.6 Hz).

^{19}F NMR (7.0 T, DMSO-d$_6$, 298 K, δ, ppm). -69.2

MS (ESI-positive ion mode): *m/z* 294.0 [M+H]$^+$.

^{1}H, ^{19}F and ^{13}C NMR Spectra of 1

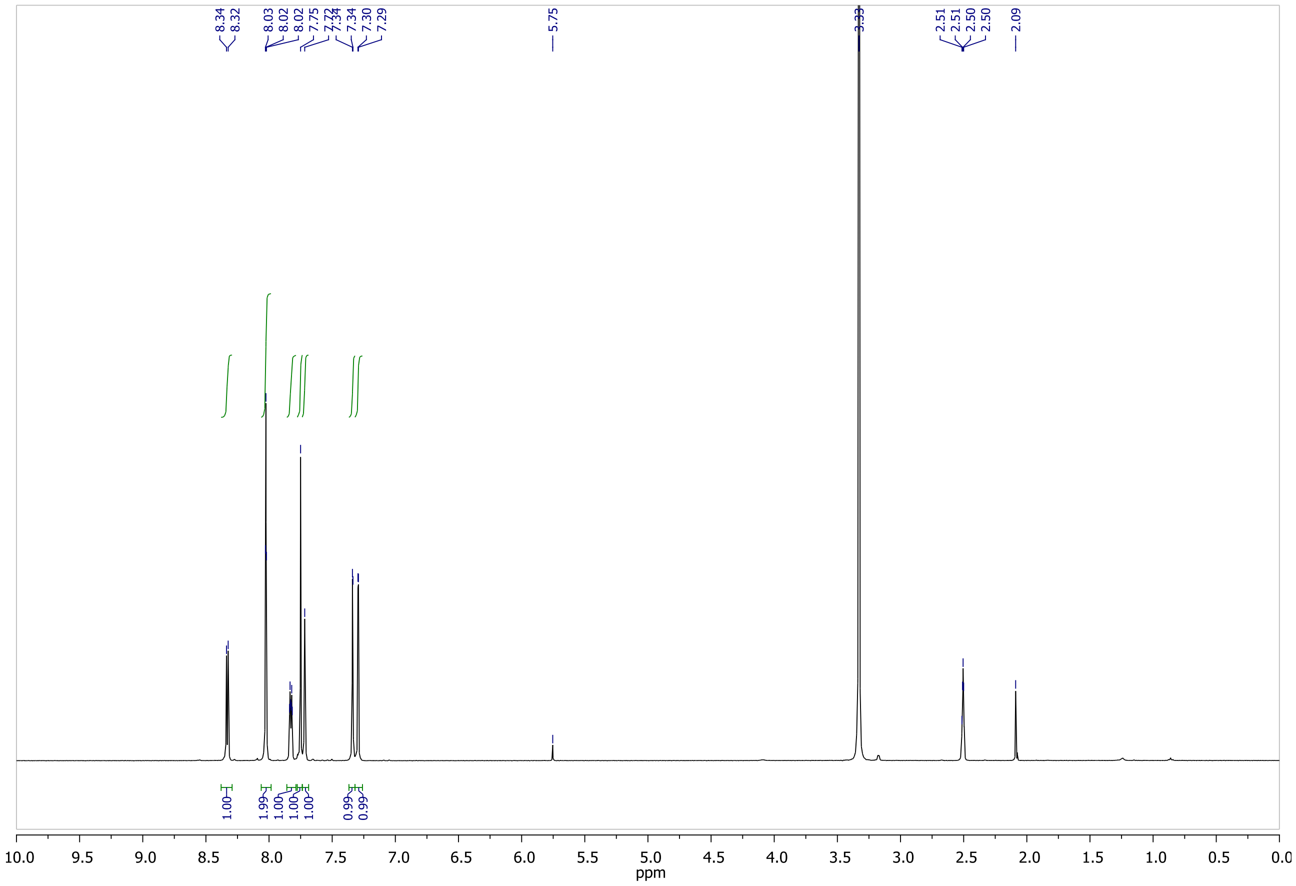

Figure S1 ^{1}H NMR of **1**, 400 MHz, DMSO-d$_6$.

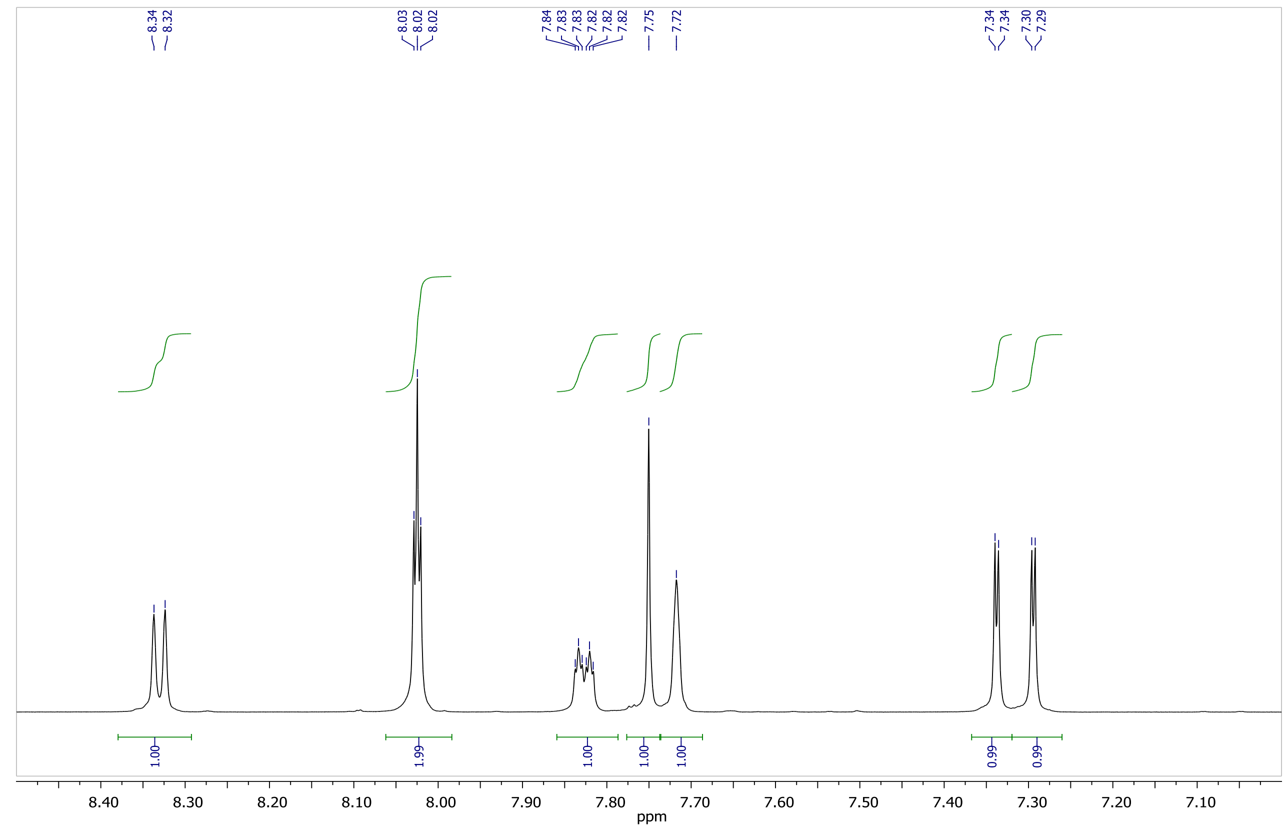

Figure S2 ^{1}H NMR of **1**, 400 MHz, DMSO-d_6, expanded region.

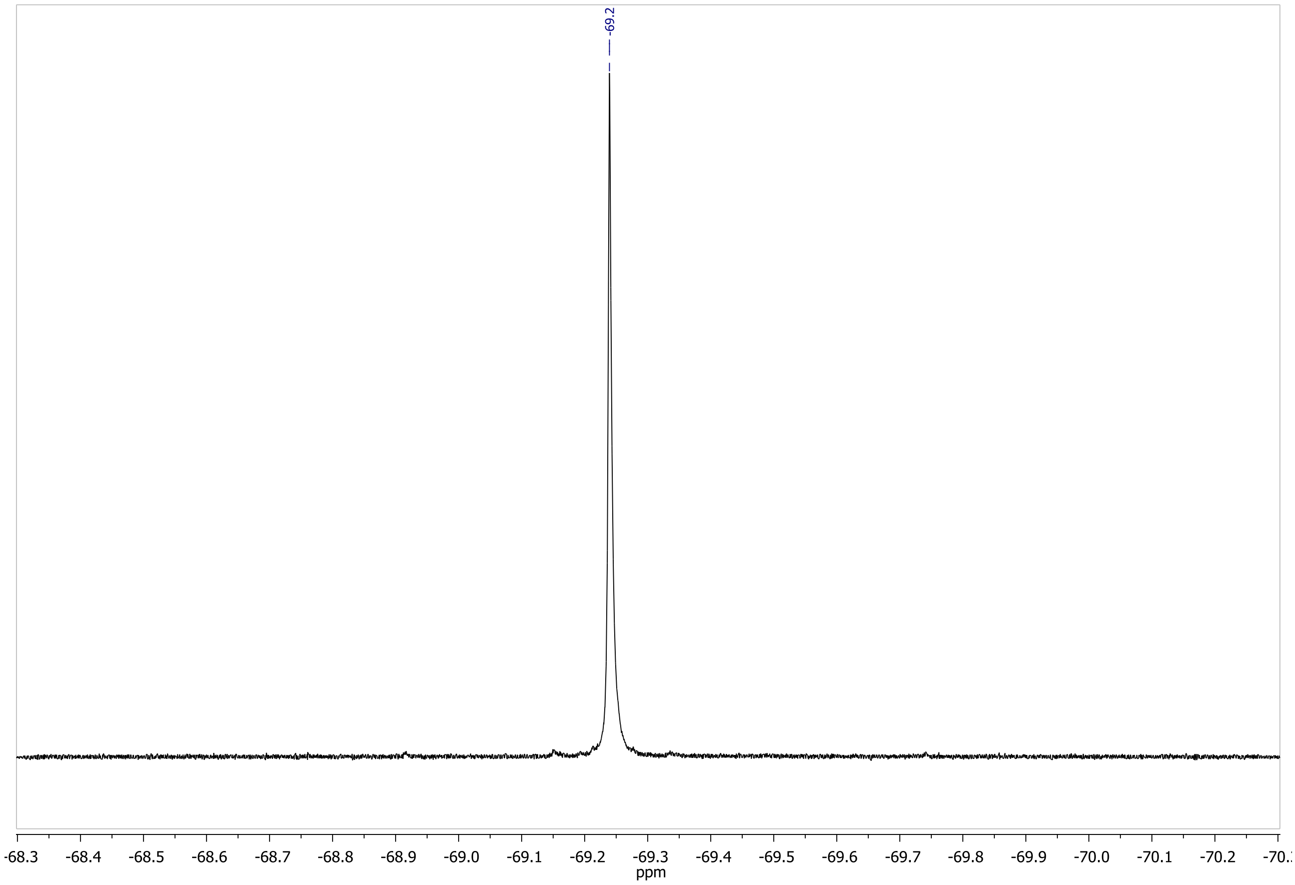

Figure S3 ^{19}F NMR of **1**, 282 MHz, DMSO-d_6.

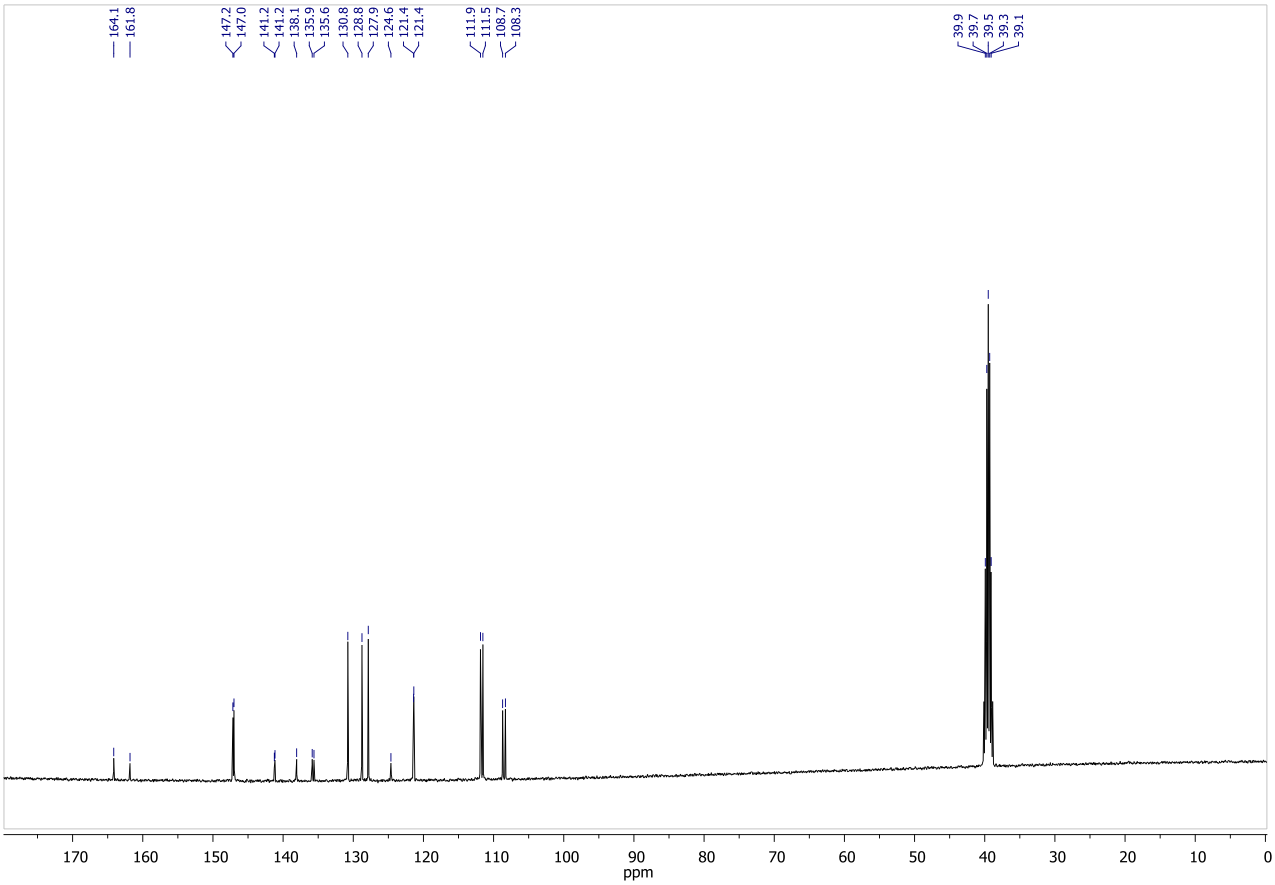

Figure S4 ^{13}C NMR of **1**, 100 MHz, DMSO-d$_6$.

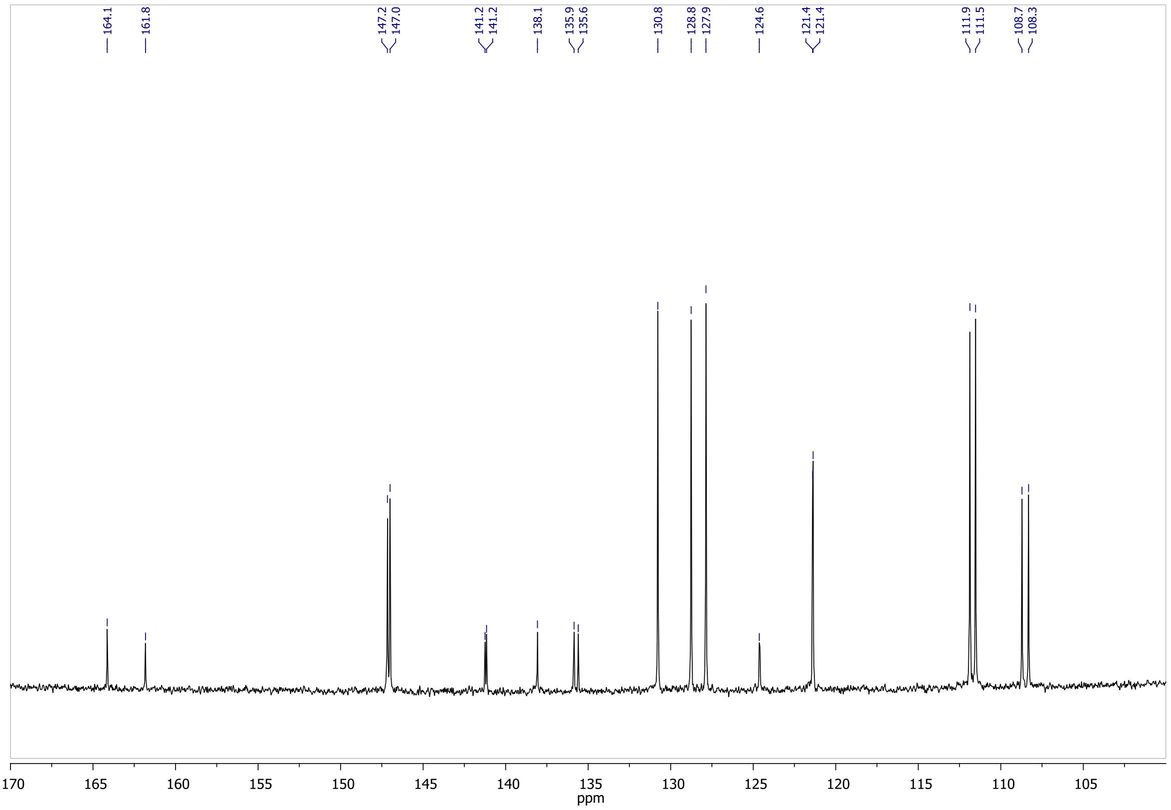

Figure S5 ^{13}C NMR of **1**, 100 MHz, DMSO-d$_6$, expanded region.

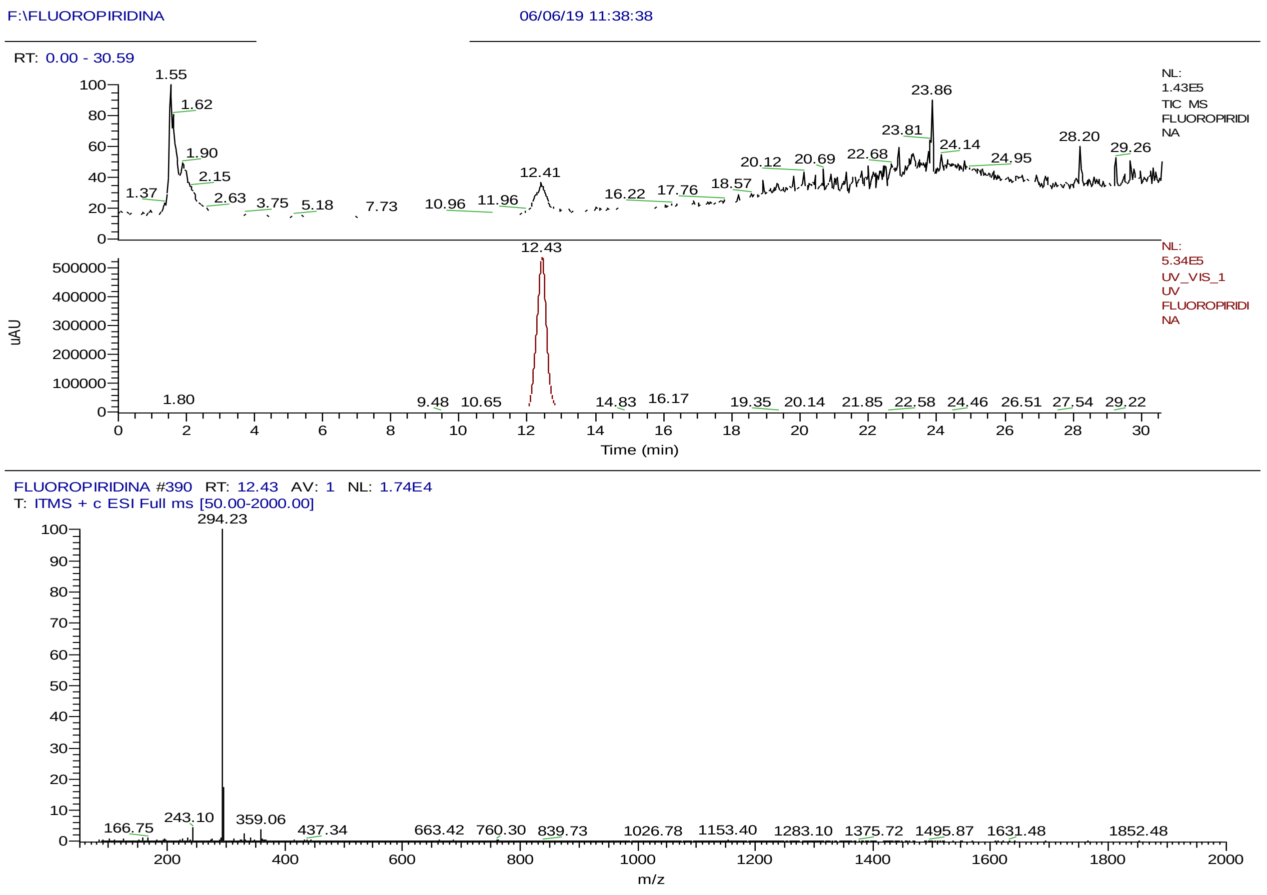

Figure S6 HPLC-MS profile of **1**.

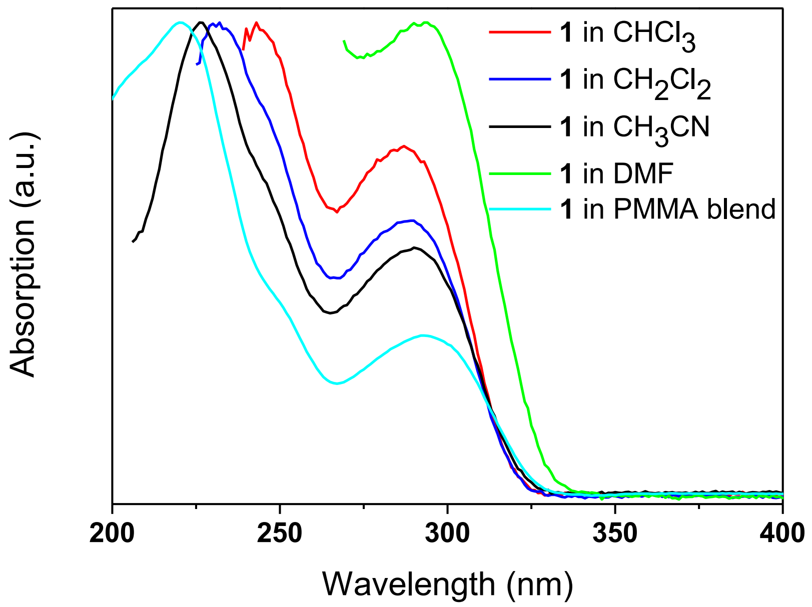

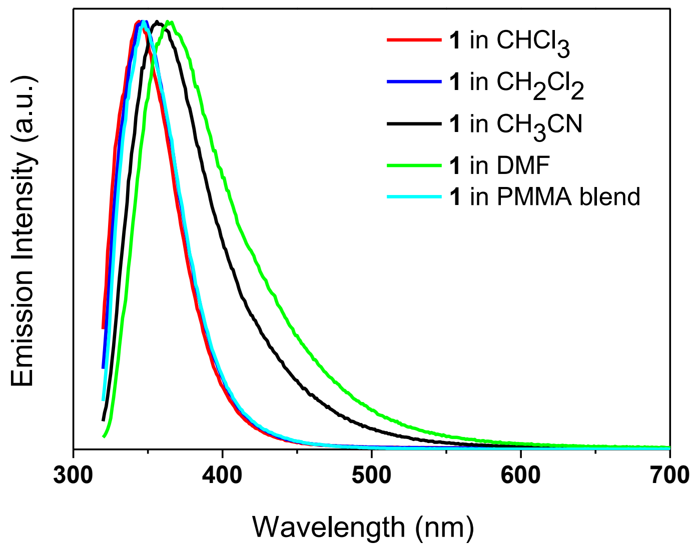

Figure S7 Normalized absorption (top) and emission (bottom) spectra of **1** in solution and PMMA blend.

Time resolved emission Spectra

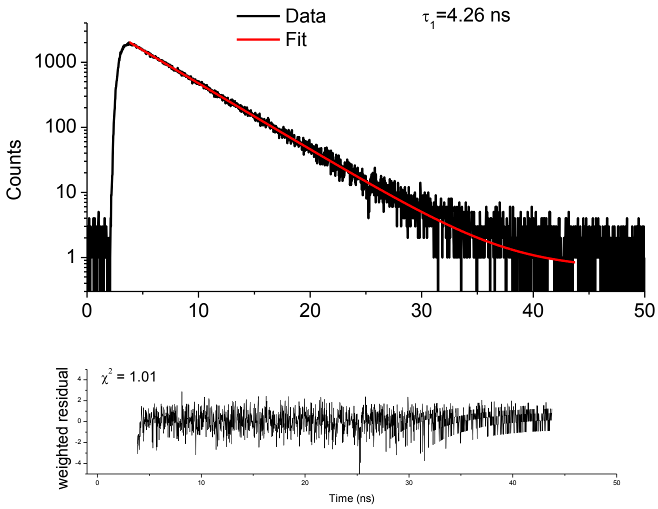

Figure S8 Emission decay of **1** in CH_3CN (10^{-5} M) at 298 K, black line (λ_{exc} 300 nm; λ_{em} 358 nm) and convolution fit (red line). Weighted residuals and χ^2 are shown under the decay curves.

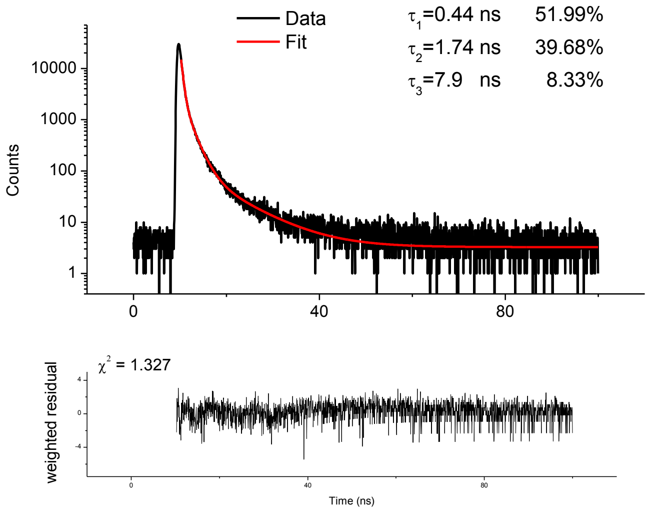

Figure S9 Emission decay of **1** in CH_3CN (10^{-5} M) at 77 K, black line (λ_{exc} 300 nm; λ_{em} 343 nm) and convolution fit (red line). Weighted residuals and χ^2 are shown under the decay curves.

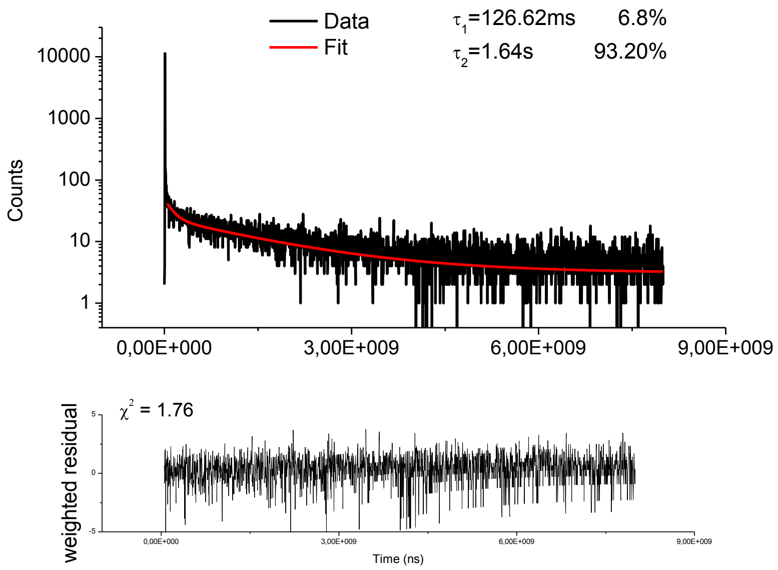

Figure S10 Emission decay of **1** in CH_3CN (10^{-5} M) at 77 K, black line (λ_{exc} 300 nm; λ_{em} 450 nm) and convolution fit (red line). Weighted residuals and χ^2 are shown under the decay curves.

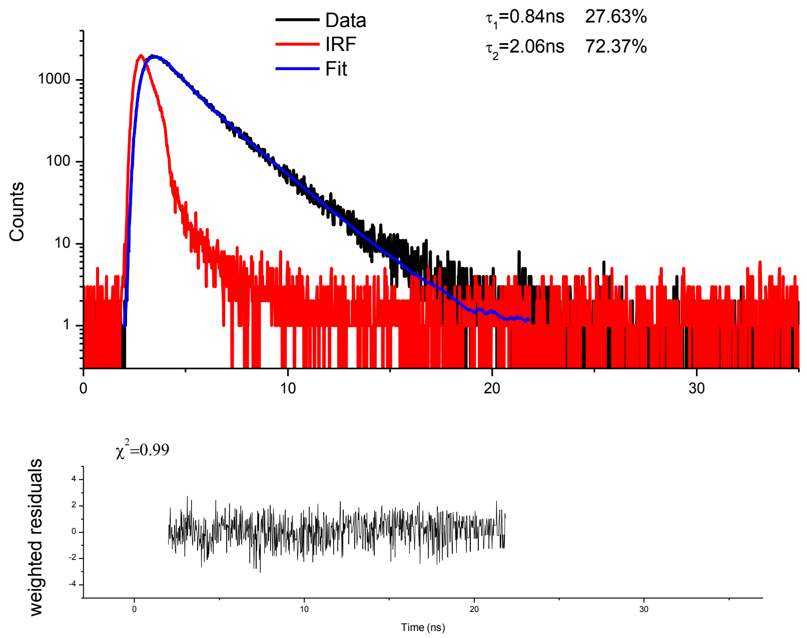

Figure S11 Emission decay of thin film **1**/PMMA at 298 K, black line (λ_{exc} 300 nm; λ_{em} 347 nm) and convolution fit (blue line). Weighted residuals and χ^2 are shown under the decay curves.

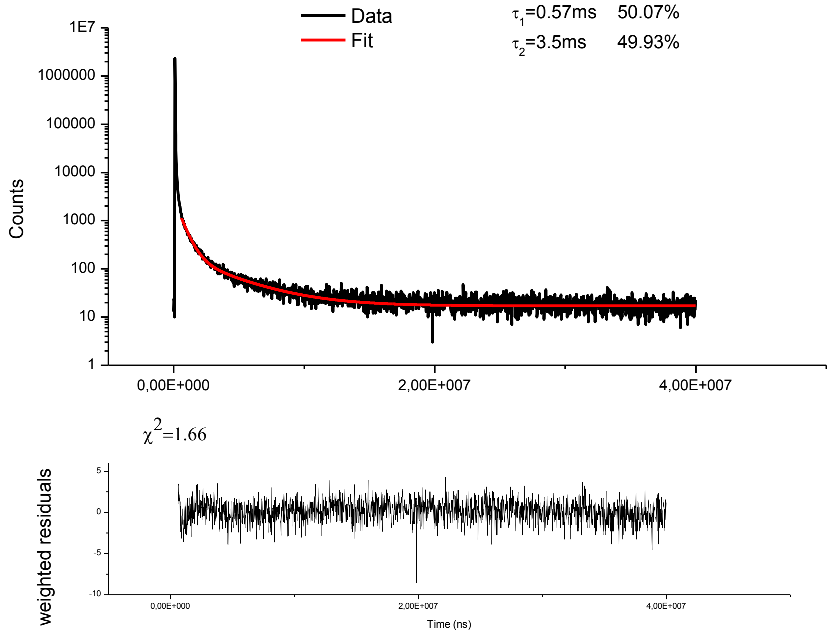

Figure S12 Emission decay of thin film **1**/PMMA at 298 K, black line (λ_{exc} 300 nm; λ_{em} 453 nm) and convolution fit (red line). Weighted residuals and χ^2 are shown under the decay curves.

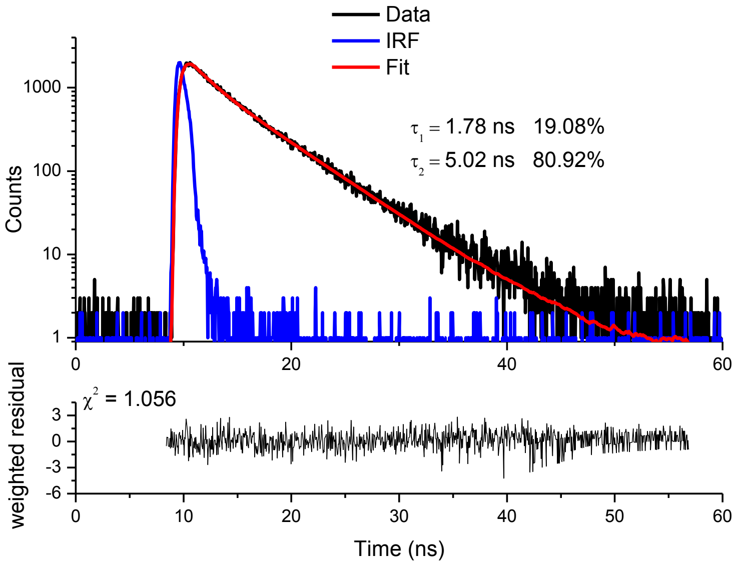

Figure S13 Emission decay of crystals of **1** at 298 K, black line (λ_{exc} 300 nm; λ_{em} 373 nm) and convolution fit (red line). Weighted residuals and χ^2 are shown under the decay curves.

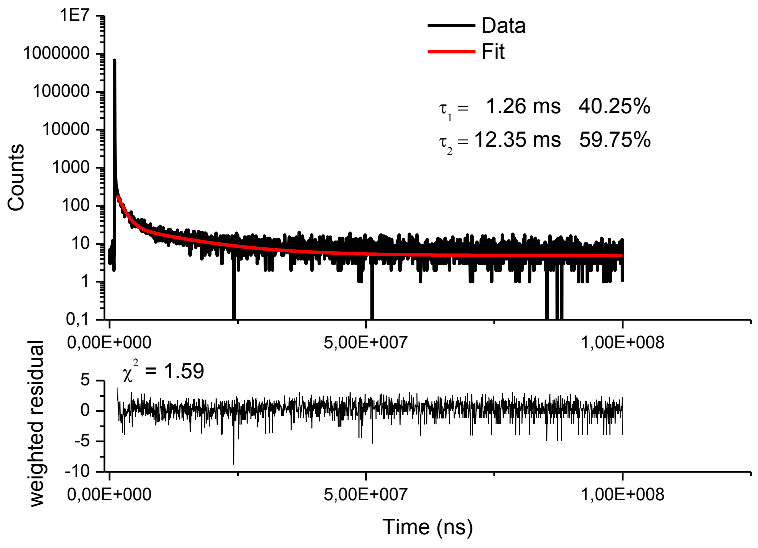

Figure S14 Emission decay of crystals of **1** at 298 K, black line (λ_{exc} 300 nm; λ_{em} 423 nm) and convolution fit (red line). Weighted residuals and χ^2 are shown under the decay curves.

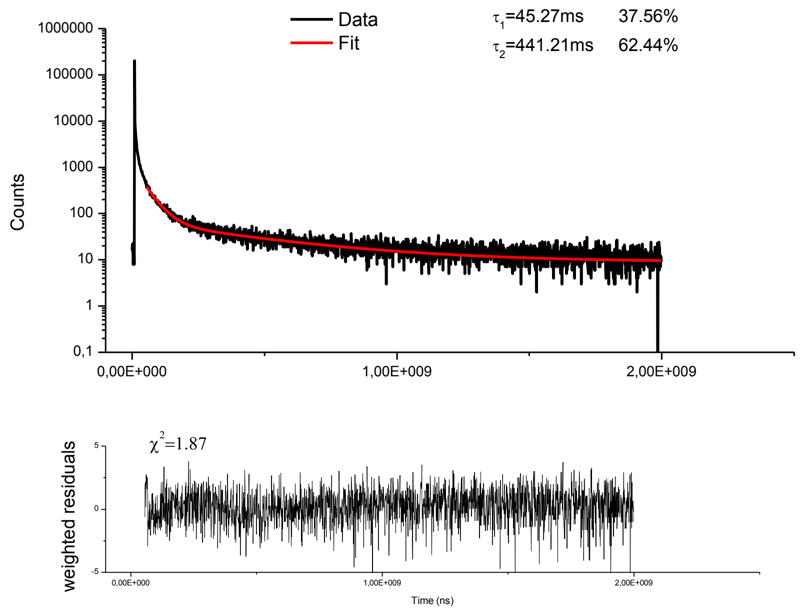

Figure S15 Emission decay of crystals of **1** at 298 K, black line (λ_{exc} 300 nm; λ_{em} 547 nm) and convolution fit (red line). Weighted residuals and χ^2 are shown under the decay curves.

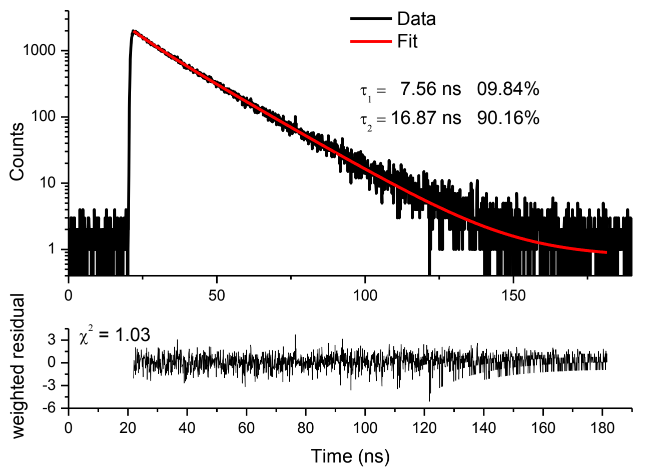

Figure S16 Emission decay of crystals of **1** at 77 K, black line (λ_{exc} 300 nm; λ_{em} 373 nm) and convolution fit (red line). Weighted residuals and χ^2 are shown under the decay curves.

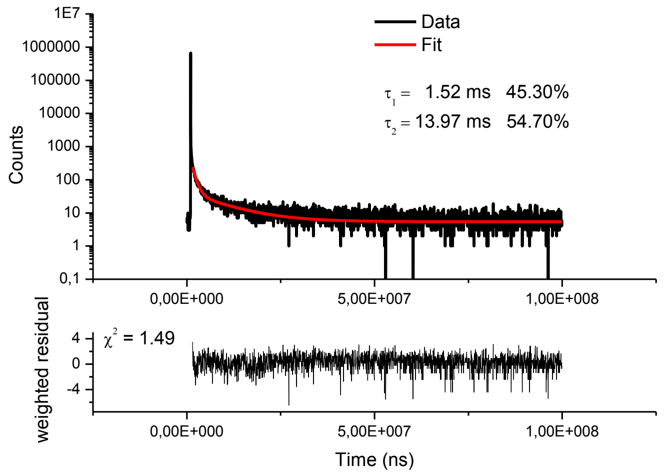

Figure S17 Emission decay of crystals of **1** at 77 K, black line (λ_{exc} 300 nm; λ_{em} 423 nm) and convolution fit (red line). Weighted residuals and χ^2 are shown under the decay curves.

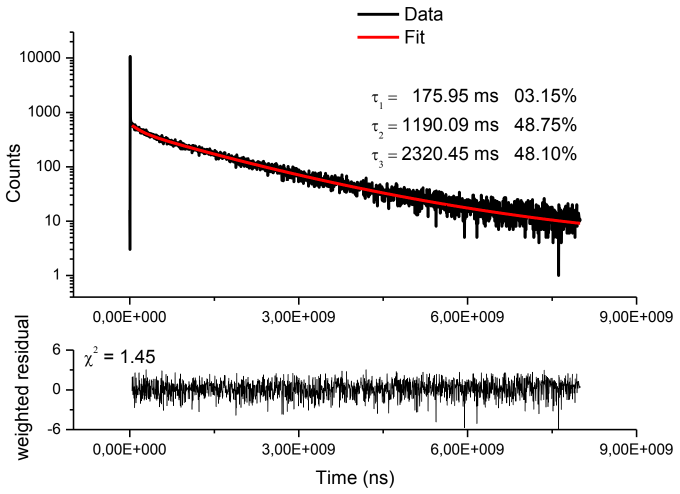

Figure S18 Emission decay of crystals of **1** at 77 K, black line (λ_{exc} 300 nm; λ_{em} 547 nm) and convolution fit (red line). Weighted residuals and χ^2 are shown under the decay curves.

Single crystal X-ray crystallographic studies

Table S1. Crystallographic data and structure refinement details for **1**.

	**1**
Chemical Formula	$C_{14}H_8FN_7$
Molecular weight	293.27
T(K)	291(2)
Crystal system	Triclinic
space group	*P*-1
a(Å)	3.8305(5)
b(Å)	11.9825(14)
c(Å)	13.8072(16)
α(°)	78.411(2)
β(°)	89.735(2)
γ(°)	87.962(2)
V(Å^3)	620.42(13)
Z	2
D_{calcd} (g cm^{-3})	1.570
μ (mm^{-1})	0.114
Crystal size (mm)	0.52 x 0.10 x 0.05
2θmax, °	61.0
No. of measured, independent and observed [I >2σ(I)] reflections	12492 / 3780 / 2772
(R_{int})/(R_σ)	0.0251 / 0.0255
data/restraints/params	3780 / 0 / 199
R[F^2 > 2σ(F^2)], wR(F^2), S	0.0484, 0.0655, 1.051
Δρmax, Δρmin (e Å^{-3})	0.341, -0.210

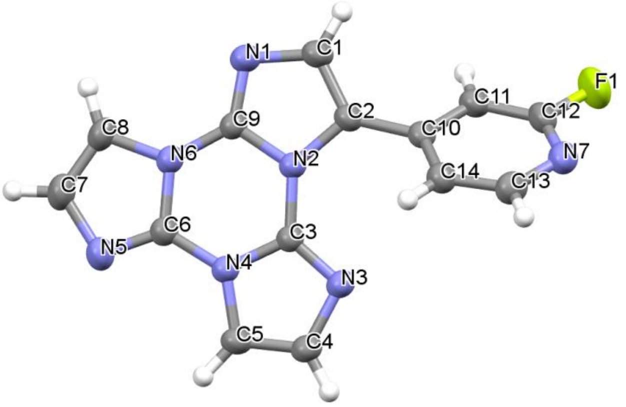

Figure S19 Ortep view of **1** with labelling scheme. Ellipsoids at 50% level of probability.

Computational studies

In Table S2 we report the first singlet and triplet excitation energies as computed in vacuo at TD-ωB97X/6-311++G(d,p) level for **1** in its optimized geometry at both the absolute and relative energy minima associated with the free rotation around the single bond connecting **TT** with 2-fluoropyridine (see Figure S21 for a plot of the scan of total energy). The corresponding simulated absorption spectrum is shown in Figure S20. The simulated spectrum is almost overlapped to the experimental one, though slightly blue-shifted, showing a major band at high energy followed by a minor band at lower energy.
A schematic representation of the computed levels for **1** and the parent cyclic triimidazole (**TT**) is shown in Figure S22, while Figure S23 displays isodensity surface plots of the frontier orbitals mainly involved in the computed transitions.

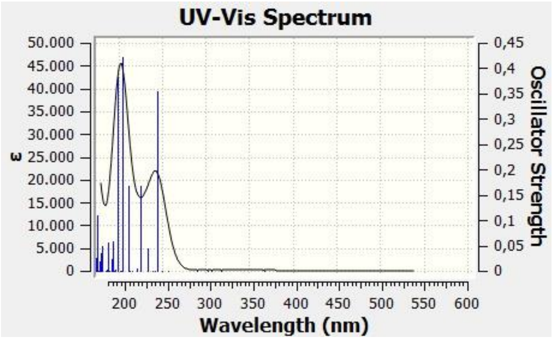

Figure S20 ωB97X/6-311++G(d,p) computed absorption spectrum of optimized **1** resulting from convolution of the excitation energies (blue sticks) with 0.25 eV of half-bandwidth.

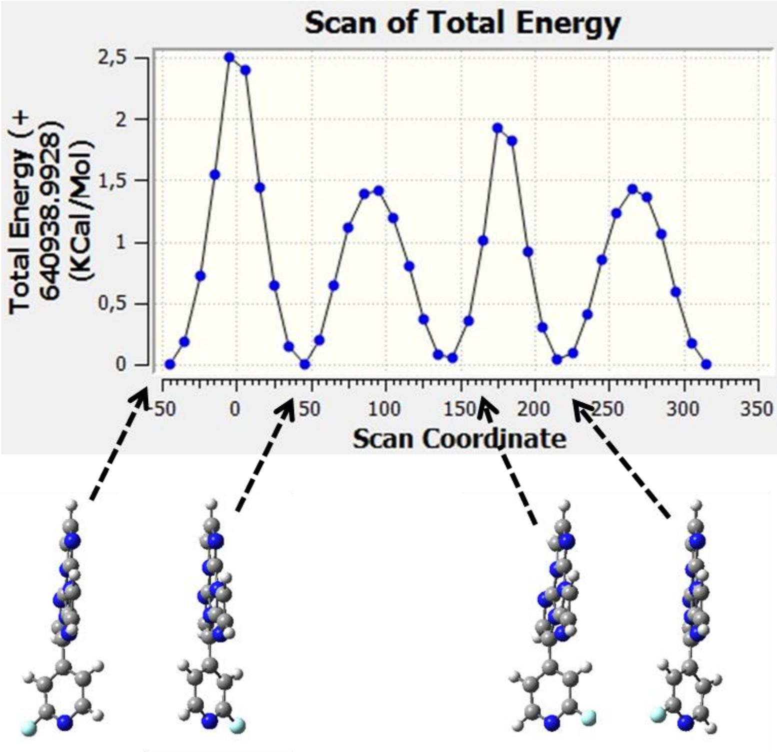

Figure S21 Scan of total energy as a function of the N2C2C10C14 torsion angle (see Figure S19).

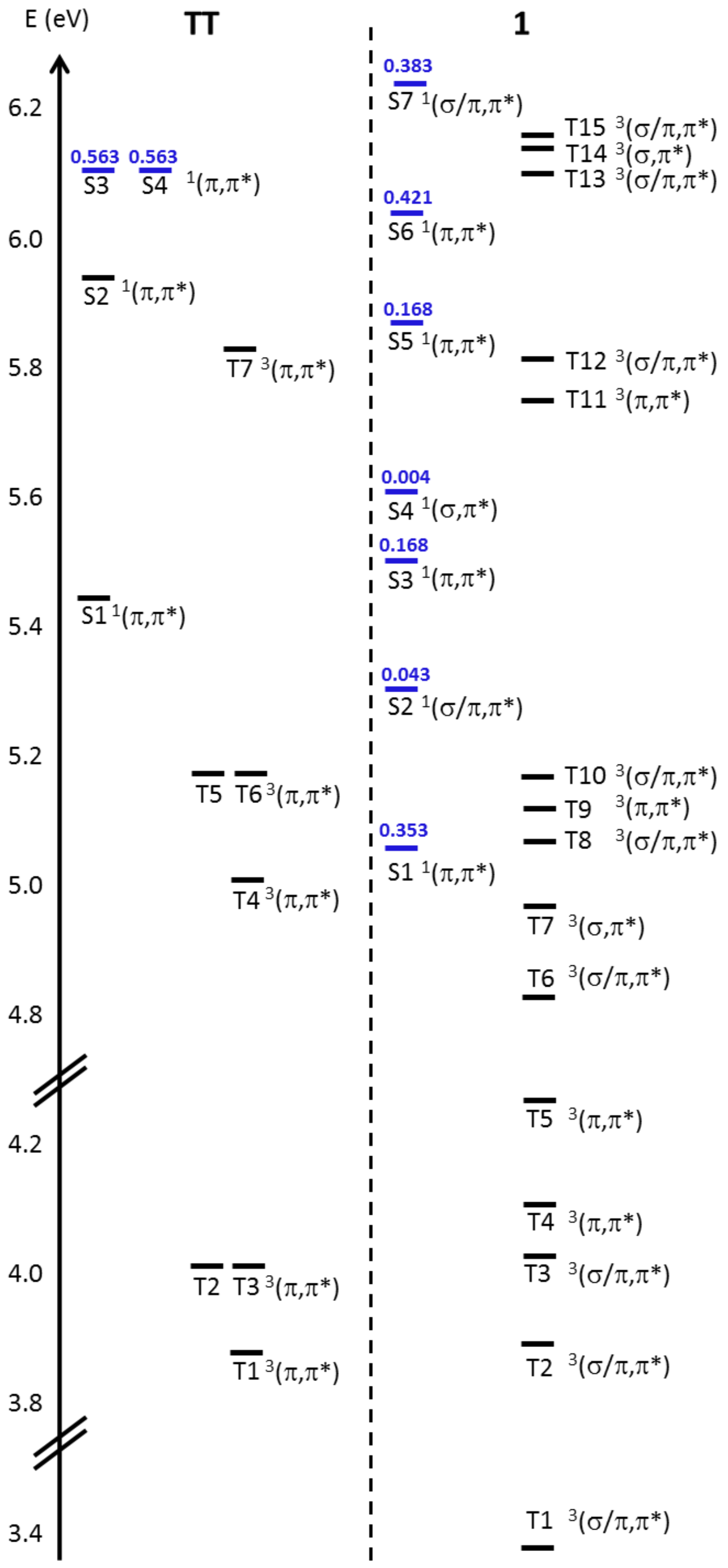

Figure S22 Electronic levels computed for **TT** (left) and **1** (right) at molecular level. In blue are reported the singlet levels with oscillator strength $f \geq 0.001$ and the corresponding values of f (see detailed information for **1** in Table S2.

Table S2 First TD-ωB97X/6-311++G(d,p) $S_0 \rightarrow S_n$ and $T_0 \rightarrow T_n$ transitions computed for **1**.

Excitation energies and oscillator strengths (absolute minimum):

```
T1  Excited State   1:      Triplet-A      3.3810 eV  366.71 nm  f=nd  <S**2>=2.000
      70 -> 76        0.15783
      72 -> 76        0.13911
      72 -> 79        0.11680
      72 -> 80       -0.10068
      72 -> 82       -0.11423
      73 -> 76       -0.13361
      74 -> 76       -0.10731
      75 -> 76        0.51831

T2  Excited State   2:      Triplet-A      3.8944 eV  318.36 nm  f=nd  <S**2>=2.000
      70 -> 76        0.16130
      72 -> 76        0.14806
      72 -> 79        0.13244
      72 -> 80       -0.11006
      72 -> 82       -0.14397
      73 -> 79        0.13784
      73 -> 80       -0.12235
      74 -> 77        0.40249
      75 -> 76       -0.14811
      75 -> 77        0.10893
      75 -> 79        0.12589
      75 -> 80       -0.11621
      75 -> 82        0.15133

T3  Excited State   3:      Triplet-A      4.0340 eV  307.34 nm  f=nd  <S**2>=2.000
      70 -> 76       -0.14513
      72 -> 76       -0.13830
      72 -> 79       -0.23063
      72 -> 80        0.20147
      73 -> 76       -0.20093
      73 -> 82        0.14803
      74 -> 76       -0.11423
      74 -> 77        0.32578
      75 -> 76        0.17987
      75 -> 77        0.12027
      75 -> 89       -0.12048

T4  Excited State   4:      Triplet-A      4.1037 eV  302.13 nm  f=nd  <S**2>=2.000
      72 -> 77       -0.17101
      73 -> 77        0.36267
      74 -> 76       -0.18971
      74 -> 77       -0.13712
      74 -> 79        0.15954
      74 -> 80       -0.14541
      74 -> 82        0.19020
      74 -> 89       -0.13752
      75 -> 77        0.22052

T5  Excited State   5:      Triplet-A      4.2670 eV  290.56 nm  f=nd  <S**2>=2.000
      72 -> 76        0.49379
      72 -> 82        0.17208
      73 -> 76        0.28047
      73 -> 82        0.15398

T6  Excited State   6:      Triplet-A      4.8307 eV  256.66 nm  f=nd  <S**2>=2.000
      70 -> 76        0.25249
      72 -> 76        0.13754
      72 -> 79       -0.10321
      72 -> 82        0.23647
      73 -> 79       -0.14035
      73 -> 80        0.12170
      75 -> 77        0.14271
      75 -> 79        0.23445
      75 -> 80       -0.21348
      75 -> 82        0.13621

T7  Excited State   7:      Triplet-A      4.9658 eV  249.68 nm  f=nd  <S**2>=2.000
      71 -> 76        0.59161
      71 -> 79        0.16326
      71 -> 80       -0.15111
      71 -> 82        0.16014
      71 -> 89        0.10271

S1  Excited State   8:      Singlet-A      5.0602 eV  245.02 nm  f=0.3533  <S**2>=0.000
      73 -> 76       -0.20656
      74 -> 77       -0.13093
      75 -> 76        0.61239

T8  Excited State   9:      Triplet-A      5.0678 eV  244.65 nm  f=nd  <S**2>=2.000
      70 -> 76       -0.10420
      73 -> 77        0.17634
      73 -> 89        0.16419
```

```
      74 -> 76         0.34580
      74 -> 79        -0.16604
      74 -> 80         0.14537
      74 -> 82        -0.14345
      75 -> 77         0.26684

T9  Excited State  10:      Triplet-A      5.1220 eV  242.06 nm  f=nd  <S**2>=2.000
      66 -> 76        -0.12080
      72 -> 76        -0.10841
      73 -> 76         0.18909
      73 -> 82        -0.12611
      74 -> 76        -0.11659
      74 -> 89         0.19439
      75 -> 77         0.33167
      75 -> 82         0.10159
      75 -> 94         0.10000
      75 -> 98        -0.10449

T10 Excited State  11:      Triplet-A      5.1745 eV  239.61 nm  f=nd  <S**2>=2.000
      70 -> 76         0.15491
      72 -> 77        -0.10478
      73 -> 76         0.13648
      73 -> 77         0.19041
      73 -> 79        -0.16713
      73 -> 80         0.14143
      73 -> 94         0.10035
      74 -> 77         0.21528
      74 -> 89         0.15306
      75 -> 82        -0.14294
      75 -> 89         0.15135
      75 -> 97         0.10108

S2  Excited State  12:      Singlet-A      5.2959 eV  234.11 nm  f=0.0435  <S**2>=0.000
      70 -> 82         0.11152
      72 -> 76         0.48505
      72 -> 82         0.12256
      73 -> 76         0.28767
      74 -> 77        -0.10480
      75 -> 76         0.11484
      75 -> 79        -0.14477
      75 -> 80         0.12363

S3  Excited State  13:      Singlet-A      5.5028 eV  225.31 nm  f=0.1683  <S**2>=0.000
      72 -> 76         0.21571
      73 -> 76        -0.18343
      74 -> 76         0.28515
      74 -> 77         0.29928
      74 -> 79        -0.14795
      74 -> 80         0.12379
      74 -> 82        -0.14262
      75 -> 76         0.14118
      75 -> 77         0.27235
      75 -> 82         0.12176

S4  Excited State  14:      Singlet-A      5.6102 eV  221.00 nm  f=0.0037  <S**2>=0.000
      71 -> 76         0.60840
      71 -> 79         0.19728
      71 -> 80        -0.17887
      71 -> 82         0.10662

T11 Excited State  15:      Triplet-A      5.7481 eV  215.70 nm  f=nd  <S**2>=2.000
      64 -> 77        -0.17076
      65 -> 76         0.12370
      73 -> 89        -0.17495
      74 -> 76         0.16815
      74 -> 94        -0.19316
      74 -> 96        -0.10716
      74 -> 97        -0.10889
      75 -> 77         0.19315
      75 -> 82        -0.17176
      75 -> 98         0.12252

T12 Excited State  16:      Triplet-A      5.8065 eV  213.53 nm  f=nd  <S**2>=2.000
      70 -> 79        -0.15420
      70 -> 80         0.12264
      70 -> 82         0.21885
      72 -> 82         0.14103
      75 -> 78        -0.16465
      75 -> 79        -0.22165
      75 -> 80         0.18736
      75 -> 81         0.11816
      75 -> 82         0.24188
      75 -> 83         0.10862

S5  Excited State  17:      Singlet-A      5.8652 eV  211.39 nm  f=0.1678  <S**2>=0.000
      74 -> 76        -0.39727
      74 -> 77         0.44035
      74 -> 79         0.13005
      74 -> 80        -0.12291
      75 -> 77        -0.11990

S6  Excited State  18:      Singlet-A      6.0372 eV  205.37 nm  f=0.4211  <S**2>=0.000
      72 -> 77        -0.13175
```

```
      73 -> 76         0.11217
      73 -> 77         0.31788
      74 -> 76        -0.21702
      75 -> 77         0.48703

T13 Excited State  19:      Triplet-A      6.0966 eV  203.37 nm  f=nd  <S**2>=2.000
      66 -> 76         0.11699
      70 -> 76         0.10033
      73 -> 76        -0.19158
      73 -> 89         0.10144
      74 -> 76        -0.17531
      74 -> 89         0.12976
      75 -> 76        -0.16002
      75 -> 77         0.23529
      75 -> 82        -0.11436

T14 Excited State  20:      Triplet-A      6.1379 eV  202.00 nm  f=nd  <S**2>=2.000
      71 -> 78        -0.18371
      71 -> 79        -0.33424
      71 -> 80         0.27364
      71 -> 81         0.14836
      71 -> 82         0.38828
      71 -> 83         0.16593
      71 -> 84         0.14518
      71 -> 86         0.15470

T15 Excited State  21:      Triplet-A      6.1578 eV  201.35 nm  f=nd  <S**2>=2.000
      64 -> 76         0.11285
      70 -> 76        -0.11916
      72 -> 76         0.10365
      73 -> 76        -0.17319
      73 -> 77         0.19364
      73 -> 94         0.13337
      73 -> 97         0.10259
      74 -> 77        -0.17037
      74 -> 89         0.20669
      74 -> 97        -0.10837
      75 -> 77        -0.11054

S7  Excited State  22:      Singlet-A      6.2359 eV  198.82 nm  f=0.3831  <S**2>=0.000
      70 -> 76         0.12562
      72 -> 79         0.15864
      72 -> 80        -0.13238
      73 -> 82        -0.10452
      74 -> 76        -0.12802
      74 -> 77        -0.26860
      75 -> 79         0.29537
      75 -> 80        -0.26372
      75 -> 81         0.11527
      75 -> 82         0.27589

S8  Excited State  23:      Singlet-A      6.2958 eV  196.93 nm  f=0.0020  <S**2>=0.000
      71 -> 78        -0.17914
      71 -> 79        -0.31261
      71 -> 80         0.25319
      71 -> 81         0.15225
      71 -> 82         0.40207
      71 -> 83         0.17026
      71 -> 84         0.14881
      71 -> 86         0.14827
```

Excitation energies and oscillator strengths (relative minimum):

```
T1   Excited State   1:      Triplet-A      3.3454 eV  370.61 nm  f=nd      <S**2>=2.000
      70 -> 76         0.14215
      72 -> 80         0.17556
      72 -> 82         0.13959
      73 -> 76        -0.19773
      74 -> 76         0.10550
      75 -> 76         0.52318

T2   Excited State   2:      Triplet-A      3.8897 eV  318.75 nm  f=nd      <S**2>=2.000
      70 -> 76        -0.14014
      72 -> 76         0.11415
      72 -> 80        -0.21775
      72 -> 82        -0.10702
      73 -> 76         0.15518
      73 -> 82        -0.10846
      74 -> 77         0.40527
      75 -> 76         0.14901
      75 -> 77        -0.10720
      75 -> 80         0.15679
      75 -> 82        -0.16451

T3   Excited State   3:      Triplet-A      4.0389 eV  306.98 nm  f=nd      <S**2>=2.000
      66 -> 76         0.10587
      70 -> 76         0.12584
      72 -> 76        -0.23095
      72 -> 80         0.21505
      72 -> 82         0.13500
      73 -> 80         0.15815
```

```
      74 -> 76        -0.12159
      74 -> 77         0.32716
      75 -> 76        -0.17105
      75 -> 77        -0.13077
      75 -> 89         0.10634

T4   Excited State   4:      Triplet-A      4.1022 eV  302.24 nm  f=nd      <S**2>=2.000
      73 -> 77         0.39222
      74 -> 76         0.18634
      74 -> 77         0.15120
      74 -> 80         0.18311
      74 -> 82        -0.22416
      74 -> 89         0.11260
      75 -> 77         0.21578

T5   Excited State   5:      Triplet-A      4.3020 eV  288.20 nm  f=nd      <S**2>=2.000
      70 -> 76         0.14068
      72 -> 76         0.55704
      72 -> 82         0.30278

T6   Excited State   6:      Triplet-A      4.8291 eV  256.74 nm  f=nd      <S**2>=2.000
      70 -> 76        -0.22445
      72 -> 80         0.23887
      72 -> 82         0.21640
      73 -> 76         0.15857
      73 -> 82         0.11261
      74 -> 77        -0.10255
      75 -> 76        -0.10018
      75 -> 77        -0.15531
      75 -> 79        -0.10322
      75 -> 80         0.22891
      75 -> 82        -0.21817

T7   Excited State   7:      Triplet-A      4.9559 eV  250.18 nm  f=nd      <S**2>=2.000
      71 -> 76         0.58526
      71 -> 80        -0.19666
      71 -> 82         0.18261

S1   Excited State   8:      Singlet-A      5.0574 eV  245.16 nm  f=0.3616  <S**2>=0.000
      73 -> 76        -0.15707
      74 -> 77         0.13988
      75 -> 76         0.62603
      75 -> 77        -0.10156

T8   Excited State   9:      Triplet-A      5.0649 eV  244.79 nm  f=nd      <S**2>=2.000
      70 -> 76         0.10208
      73 -> 77        -0.20637
      73 -> 88        -0.10228
      73 -> 89        -0.15034
      73 -> 90         0.10601
      74 -> 76         0.36033
      74 -> 80         0.15963
      74 -> 82        -0.17677
      74 -> 96        -0.12118
      75 -> 77        -0.22793
      75 -> 80        -0.10613

T9   Excited State  10:      Triplet-A      5.1209 eV  242.11 nm  f=nd      <S**2>=2.000
      66 -> 76        -0.11536
      73 -> 76         0.22452
      73 -> 80         0.11613
      73 -> 82        -0.13795
      74 -> 88        -0.10677
      74 -> 89        -0.16235
      74 -> 90         0.11538
      75 -> 77         0.35479
      75 -> 98        -0.12314

T10  Excited State  11:      Triplet-A      5.1790 eV  239.40 nm  f=nd      <S**2>=2.000
      64 -> 76         0.11800
      70 -> 76         0.17098
      72 -> 80        -0.11190
      73 -> 76         0.12507
      73 -> 77         0.21722
      73 -> 80         0.14502
      73 -> 82        -0.16246
      74 -> 77        -0.21101
      74 -> 89        -0.11255
      75 -> 82        -0.15501
      75 -> 89         0.12473
      75 -> 90        -0.11290

S2   Excited State  12:      Singlet-A      5.2862 eV  234.54 nm  f=0.0483  <S**2>=0.000
      70 -> 80        -0.11084
      72 -> 76         0.57636
      72 -> 80        -0.14287
      72 -> 82         0.14495
      73 -> 76         0.12052
      75 -> 80        -0.13062
      75 -> 82        -0.10081

S3   Excited State  13:      Singlet-A      5.4783 eV  226.32 nm  f=0.1199  <S**2>=0.000
      73 -> 76         0.27437
```

```
      74 -> 76         0.30123
      74 -> 77         0.30435
      74 -> 80         0.16029
      74 -> 82        -0.17887
      75 -> 76        -0.10633
      75 -> 77        -0.26577
      75 -> 80         0.10024
      75 -> 82        -0.12996

S4   Excited State  14:      Singlet-A      5.6097 eV  221.02 nm  f=0.0267  <S**2>=0.000
      71 -> 76         0.59499
      71 -> 79         0.10062
      71 -> 80        -0.23125
      71 -> 82         0.12473

T11  Excited State  15:      Triplet-A      5.7016 eV  217.45 nm  f=nd      <S**2>=2.000
      70 -> 80         0.18983
      70 -> 82         0.18302
      73 -> 80        -0.12312
      73 -> 82        -0.17735
      75 -> 78         0.13979
      75 -> 80         0.22434
      75 -> 82         0.33708

T12  Excited State  16:      Triplet-A      5.7611 eV  215.21 nm  f=nd      <S**2>=2.000
      64 -> 77         0.17084
      65 -> 76         0.13618
      70 -> 76         0.10880
      70 -> 82         0.11636
      73 -> 89        -0.16084
      73 -> 90         0.12987
      74 -> 76        -0.16330
      74 -> 93         0.11092
      74 -> 95         0.12495
      74 -> 96        -0.16313
      75 -> 77         0.15727
      75 -> 80         0.20103
      75 -> 97         0.10674
      75 -> 98         0.10985

S5   Excited State  17:      Singlet-A      5.8579 eV  211.65 nm  f=0.1760  <S**2>=0.000
      73 -> 76         0.11402
      73 -> 82        -0.10516
      74 -> 76        -0.39561
      74 -> 77         0.44700
      74 -> 80        -0.11574
      74 -> 82         0.12398
      75 -> 77         0.11587
      75 -> 82        -0.10960

S6   Excited State  18:      Singlet-A      6.0311 eV  205.58 nm  f=0.4024  <S**2>=0.000
      73 -> 76         0.12742
      73 -> 77         0.33256
      74 -> 76         0.21216
      75 -> 77         0.49634

T13  Excited State  19:      Triplet-A      6.0895 eV  203.60 nm  f=nd      <S**2>=2.000
      66 -> 76         0.11441
      67 -> 77         0.10761
      73 -> 76        -0.21300
      73 -> 80        -0.10767
      73 -> 82         0.11004
      74 -> 76         0.17630
      74 -> 89        -0.11575
      75 -> 76        -0.15981
      75 -> 77         0.23597
      75 -> 82        -0.12927

T14  Excited State  20:      Triplet-A      6.1387 eV  201.97 nm  f=nd      <S**2>=2.000
      71 -> 78         0.16931
      71 -> 79        -0.16986
      71 -> 80         0.41282
      71 -> 82         0.42331
      71 -> 83         0.10329
      71 -> 84        -0.15203
      71 -> 86        -0.14554

T15  Excited State  21:      Triplet-A      6.1527 eV  201.51 nm  f=nd      <S**2>=2.000
      64 -> 76        -0.10096
      67 -> 77        -0.11360
      70 -> 76        -0.12854
      73 -> 76        -0.18667
      73 -> 77         0.21542
      73 -> 95         0.10482
      73 -> 96        -0.15310
      74 -> 77         0.16898
      74 -> 89        -0.16854
      74 -> 90         0.12305
      74 -> 96        -0.13412
      75 -> 77        -0.11821
      75 -> 80        -0.10886
      75 -> 82         0.11291
```

```
S7   Excited State  22:      Singlet-A      6.2178 eV  199.40 nm  f=0.4217  <S**2>=0.000
      70 -> 76        -0.12127
      71 -> 82        -0.10851
      72 -> 80        -0.14468
      72 -> 82        -0.13484
      73 -> 80        -0.11976
      74 -> 76        -0.13011
      74 -> 77        -0.25303
      75 -> 78         0.21692
      75 -> 79        -0.16553
      75 -> 80         0.33679
      75 -> 82        -0.23256

S8   Excited State  23:      Singlet-A      6.2809 eV  197.40 nm  f=0.0260  <S**2>=0.000
      71 -> 78         0.15619
      71 -> 79        -0.15203
      71 -> 80         0.37194
      71 -> 82         0.41255
      71 -> 84        -0.14017
      71 -> 86        -0.13711
      75 -> 82        -0.17426
```

Figure S23 Isodensity surface plot of the frontier orbitals of **1** mainly involved in the computed transitions (isosurface values: 0.03, energies in a.u).

<u>Highest Occupied MOs</u>

MO	E(a.u.)	symmetry
70	-0.41040	mixed σ/π
71	-0.39524	σ
72	-0.36515	π
73	-0.35931	π
74	-0.34069	π
75	-0.33351	π

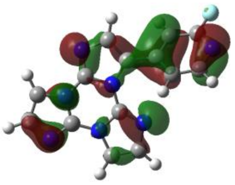
70

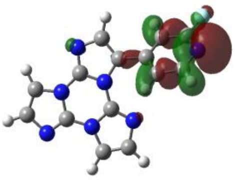
71

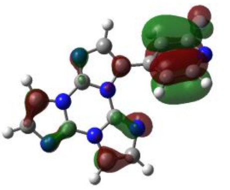
72

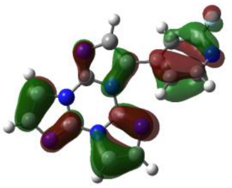
73

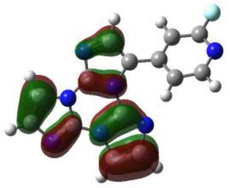
74

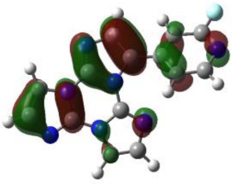
75

<u>Lowest Unoccupied MOs</u>

MO	E(a.u.)	symmetry
76	0.00831	π
77	0.03787	π
78	0.03972	σ
79	0.04677	π
80	0.04893	π
81	0.05027	σ
82	0.05650	π

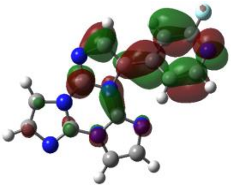
76

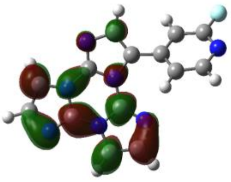
77

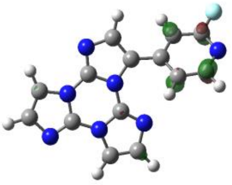
78

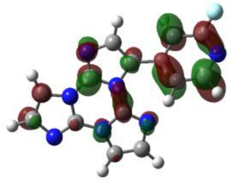
79

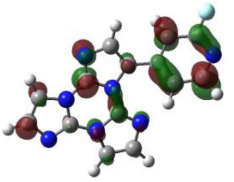
80

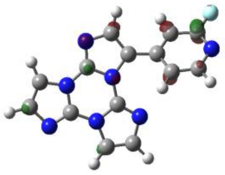
81

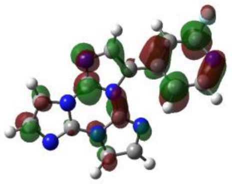
82

Cyclic voltammetry

Cyclic voltammetry patterns for **1** were recorded on a glassy carbon (GC) working electrode (Metrohm, 1 mm diameter) using a platinum wire as counter electrode and an aqueous saturated calomel electrode (SCE) as reference one inserted in a separated compartment filled with the working medium and ending with a porous frit to avoid leakage of water and chloride anions. The experiments were carried out in dimethylformamide at 1.3×10^{-3} M concentration of the complex in the presence of tetrabutylammonium hexafluorophosphate salt (0.1 M) as a supporting electrolyte, removing oxygen by nitrogen bubbling. Potentials were referred to the Fc^{+}|Fc intersolvental redox couple, signals were recorded under the same conditions. Ohmic drop correction was applied by the positive feedback method.

Table S3 Selected CV data obtained on GC electrode, in DMF with 0.1 M $TBAPF_6$, at a scan rate of 200 mVs^{-1}, potentials are referred to the Fc^{+}|Fc redox couple. The HOMO and LUMO energy levels were calculated with the *onset criterion* using the equation $E_{LUMO\ (onset)}$ (eV) = -1e × [($E_{red,\ onset}$ V(Fc^{+}|Fc) + 4.8 V(Fc^{+}|Fc *vs* zero)] and $E_{HOMO\ (onset)}$ (eV) = -1e × [($E_{ox,\ onset}$ /V(Fc^{+}|Fc) + 4.8 V(Fc^{+}|Fc *vs* zero)] respectively. The bandgap was determined from the equation $E_{g\ (onset)}$ (eV) = ($E_{LUMO\ (onset)}$ − $E_{HOMO\ (onset)}$)

	$E_{ox,\ onset}$ /V *vs* Fc^{+}|Fc	$E_{red,onset}$ /V *vs* Fc^{+}|Fc	E_{HOMO} (onset) /eV	E_{LUMO}, (onset) /eV	E_g (onset) /eV
**1**	1,16	- 2.36	- 5.96	- 2.44	3.52

Figure S24 Cyclic voltammetry patterns of **1** recorded on GC electrode at 0.2 V s^{-1} in DMF+$TBAPF_6$ 0.1 M

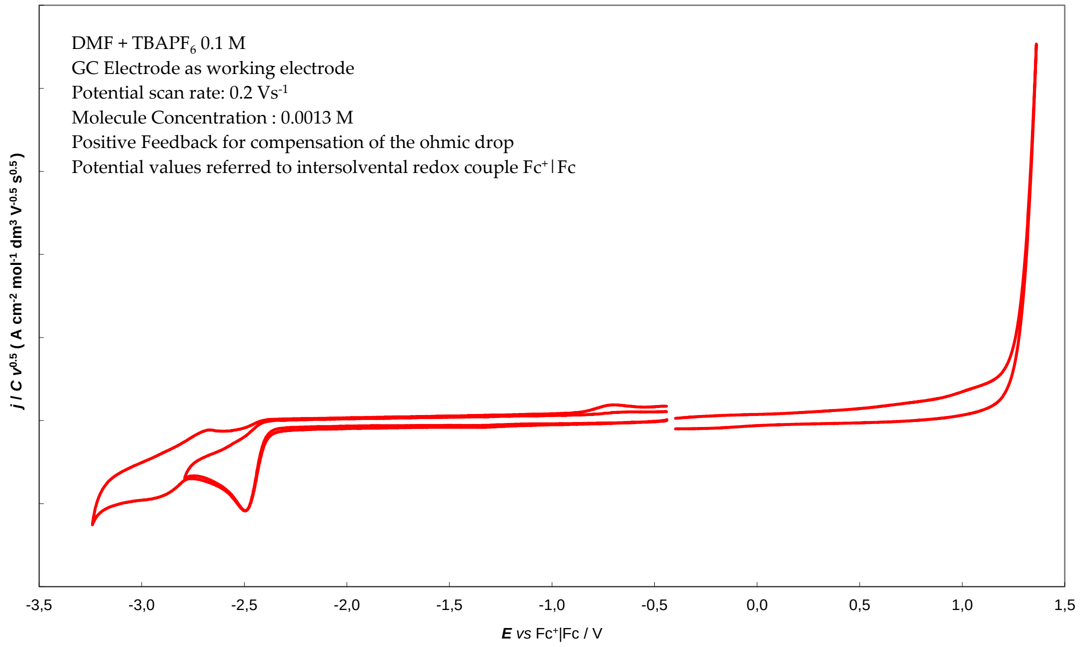

Supplement: Supplementary file 1 [file molecules-24-02552-s001.zip › molecules-535141-SI/supplementary files revised/ESI.pdf]
